# TERT expression attenuates metabolic disorders in obese mice by promoting adipose stem and progenitor cell expansion and differentiation

**DOI:** 10.1016/j.molmet.2025.102262

**Published:** 2025-10-03

**Authors:** Laura Braud, Manuel Bernabe, Julien Vernerey, Antonio M.A. Miranda, Andrea Dominguez, Dmitri Churikov, Manon Richaud, Frédéric Jourquin, Liam Mc Allan, Christophe Lachaud, Jesus Gil, Will Scott, Vincent Géli

**Affiliations:** 1 Marseille Cancer Research Centre (CRCM), U1068 INSERM, UMR7258 CNRS, UM105 Aix-Marseille University, Institut Paoli-Calmettes, Marseille, France; 2 Marseille Cancer Research Centre (CRCM), U1068 INSERM, UMR7258 CNRS, UM105 Aix-Marseille University, Institut Paoli-Calmettes, CRCM CoreTech, Marseille, France; 3 MRC Laboratory of Medical Sciences (LMS), Du Cane Road, London W12 0NN, UK; 4 Institute of Clinical Sciences (ICS), Faculty of Medicine, Imperial College London, Du Cane Road, London W12 0NN, UK; 5 Institute for Research on Cancer and Aging of Nice (IRCAN), INSERM, Université Côte d’Azur, Centre National de la Recherche Scientifique (CNRS), Nice, France

**Keywords:** Adipose-stem-cells, Telomeres, Obesity, Senescence, Telomerase

## Abstract

**Background and aims:**

Adipose tissue (AT) senescence, induced by obesity or aging, leads to a reduced capacity for tissue remodeling and a chronic pro-inflammatory state, which leads to the onset of metabolic pathologies. Cellular senescence is triggered by various stresses, in particular excessive shortening of telomeres, which activates the p21 pathway and leads to the arrest of the cell cycle. We used the mouse model p21^+/Tert^ expressing TERT from the Cdkn1a locus to investigate whether counteracting telomere shortening by telomerase (TERT) specifically in pre-senescent cells could improve obesity-induced metabolic disorders.

**Results:**

Our study demonstrates that conditional expression of TERT reduces insulin-resistance and glucose intolerance associated with obesity. In AT, this is accompanied by a decrease in the number of senescent p21-positive cells, very short telomeres, and oxidative DNA damage. Single nucleus RNA-seq data reveal TERT expression attenuates senescence induced by HFD in particular in adipose stem and progenitor cells (ASPC). We demonstrate that ASPC expansion and differentiation are promoted in p21^+/Tert^ obese mice, thereby improving AT plasticity. Furthermore, we show that TERT expression enhances mitochondrial function and alleviates oxidative stress in ASPC. This process contributes to the AT hyperplasia with increased number of adipocytes which has been shown to have a protective effect against obesity-associated metabolic disorders.

**Conclusions:**

These results underscore TERT's role in mitigating obesity-related metabolic dysfunction. Conditional TERT expression may therefore represent as a promising therapeutic strategy for obesity-associated metabolic disorders.

## Highlights

• mTERT expression from p21 promoter reduces metabolic disorders in HFD-fed male mice.
• Conditional mTERT expression attenuates obesity-associated telomere shortening and oxidative damage in the adipose tissue.
• Senescence is attenuated by the conditional expression of mTERT.
• mTERT supports ASPC expansion and differentiation in HFD-fed mice.

## Graphical abstract

**Figure:**
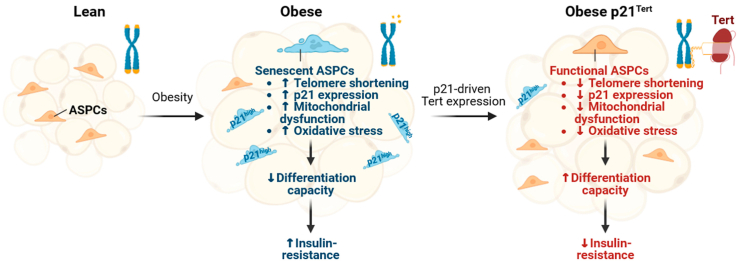

## 1 Introduction

Obesity has increased dramatically in recent decades and is associated with increased risks of insulin-resistance, type 2 diabetes, cardiovascular diseases and cancer [1]. Obesity is characterized by adipose tissue (AT) expansion, a crucial organ in the pathology due to its fat storing capacity as well as its endocrine function, promoted by the secretion of several factors and hormones that influence whole body metabolism [2,3]. During obesity, numerous events participate in AT dysfunction, including adipocyte hypertrophy, impaired adipose stem and progenitor cell (ASPC) differentiation capacity, pro-inflammatory immune cell infiltration and fibrosis [4]. ASPCs encompass both early progenitors (Adipose Stem Cells, ASCs) and more committed progenitor cells. ASCs are identified as a subpopulation of early adipocyte progenitor cells (CD29^+^; CD34^+^; Sca-1^+^; CD24^+^) residing in the vascular niche of adipose tissue (stromal vascular fraction, SVF) [5,6]. As differentiation progresses, ASCs lose CD24 expression and become further committed to the adipocyte lineage, giving rise to a CD24^-^ pre-adipocyte population that ultimately matures into adipocytes [5,6]. Therefore, ASPCs confer a high degree of plasticity to the AT. There are distinct subtypes of ASPC in AT reflecting the complexity of ASPC in different adipose deposits [7]. Obesity-induced progenitor dysfunction such as senescence and reduced adipogenic properties, lead to restricted capacity of AT remodeling and contribute to obesity associated metabolic disorders [8].

Numerous studies in humans have shown that obesity is associated with shortening of telomeres [[9], [10], [11]]. This correlation between reduced telomere length and obesity is probably mediated by the oxidative stress and inflammation associated with obesity that can damage telomeres [[12], [13], [14]]. Recent studies in mice demonstrated that specific inactivation of TERT (the catalytic subunit of mouse telomerase) in mouse ASPCs results in premature telomere shortening and cellular senescence accompanied by adipocyte hypertrophy, inflammation, fibrosis and systemic insulin resistance [13]. However, TERT expression or increased activity during obesity has generally not been studied in humans or mice. Recently, a study conducted on mice revealed that a rare population of adipose stem cells expressing the *Tert* gene had the ability to self-renew and could differentiate into mature adipocytes in response to a high-fat diet [15].

It has been shown that in mice made obese by High Fat Diet (HFD), p21^Cdkn1a^ is highly expressed in visceral AT, with p21^high^ cells being mainly ASPCs, endothelial cells, macrophages and leukocytes [16]. Clearance of these p21^high^ senescent cells in obese mice also alleviated AT dysfunctions and insulin-resistance [16]. Collectively, these studies revealed that senescent cells instigate obesity associated metabolic disorders [17]. Importantly, adipocytes from mice fed a high-fat diet show higher levels of oxidative DNA damage before the onset of alterations in adipocyte insulin sensitivity and glucose homeostasis [17]. This is evidenced by an increase in cells with β-galactosidase activity, increased ATP content, the occurrence of DNA damage, and telomere attrition contributing to the activation of the Tp53-p21 pathway [18,19].

In humans, cellular senescence also increases in obese patients, as well as in patients suffering from type 2 diabetes or non-alcoholic fatty liver disease [20]. Accumulation of senescent cells within AT is the main contributor to its dysfunctions [14], and obesity-induced enlargement of adipocytes positively correlates with senescence [21]. Although elimination of cells expressing high levels of p21 and p16 reduced AT inflammation and improved insulin sensitivity in obese mice, much less is known about the consequences of telomere shortening and the role of TERT in AT function.

We recently generated p21^+/Tert^ transgenic mouse models in which telomerase reverse transcriptase (Tert) is expressed from the p21^Cdkn1a^ promoter [22]. We found that such expression of TERT reduced p21 levels, oxidative damage, endothelial cells (ECs) senescence in lungs in p21^+/Tert^ old mice and attenuated senile emphysema [22]. We sought to enforce TERT expression specifically in p21-expressing cells, notably in pre-senescent cells including cells with dysfunctional telomeres. We have taken advantage of these mouse models to study the impact of counteracting telomere attrition on obesity-induced metabolic disorders in mice fed with high fat diet (HFD). We demonstrate that TERT expression driven by the p21 promoter improves insulin sensitivity and glucose tolerance in obese mice. This improvement is associated with a reduction in p21-expressing cells, a decrease in critically short telomeres, and an attenuation of cellular senescence typically observed in HFD-fed control mice. Additionally, TERT expression in ASPCs reduces oxidative stress. In the adipose tissue of obese p21^+/Tert^ mice, TERT promotes ASPCs proliferation and differentiation into mature adipocytes. Together, these findings reveal that TERT alleviates metabolic disorders primarily by suppressing cellular senescence, reducing oxidative damage, and enhancing ASPC expansion and adipogenic differentiation.

## 2 Results

### 2.1 p21 promoter-dependent expression of TERT prevents obesity-associated metabolic disorders in male obese mice

**Figure 1:**
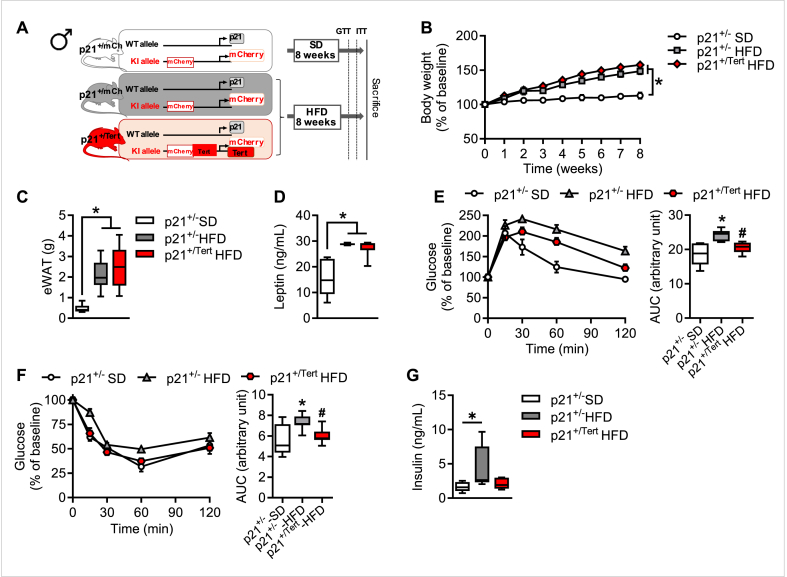
**p21 promoter-driven Tert expression alleviates obesity-associated metabolic disorders** A. Schematic representation of mouse models (High Fat Diet, HFD, was given for 8 weeks) B. Weight acquisition kinetics of the indicated HFD mice (n = 7 p21^+/−^ SD, n = 19 p21^+/−^ HFD and n = 18 p21^+/Tert^ HFD mice) C. Epididymal white adipose tissue (eWAT) weight of HFD mice at sacrifice (n = 6 p21^+/−^ SD, n = 7 p21^+/−^ HFD and n = 7 p21^+/Tert^ HFD mice) D. Leptin level in the blood plasma measured by Leptin ELISA (n = 6 p21^+/−^ SD, n = 5 p21^+/−^ HFD and n = 6 p21^+/Tert^ HFD mice) E. Left, Glucose Tolerance Test (GTT) was performed by an intraperitoneal injection of glucose (1.5 g/kg) and measurement of glycemia via tail clip (Caresens® N, DinnoSanteTM) at different time points. Right, area under the curve (AUC) of the GTT (n = 6 p21^+/−^ SD, n = 6 p21^+/−^ HFD and n = 7 p21^+/Tert^ HFD mice) F. Left, Insulin Tolerance Test (ITT) was performed by an intraperitoneal injection of insulin (0.3 UI/kg) and glucose was measured via tail clip (Caresens® N, DinnoSanteTM) at different time points. Right, area under the curve (AUC) of the ITT (n = 7 p21^+/−^ SD, n = 9 p21^+/−^ HFD and n = 11 p21^+/Tert^ HFD mice) G. Plasmatic insulin level (n = 5 p21^+/−^ SD, n = 5 p21^+/−^ HFD and n = 6 p21^+/Tert^ HFD mice) All data are shown as mean ± SEM. ∗p < 0.05 vs. p21^+/−^ SD mice (white bars), ^#^p < 0.05 vs. p21^+/−^ HFD (grey bars). Student's t test or one-way ANOVA with Fisher multiple comparison test.

Because p21 is highly induced in mice fed with HFD [16], we evaluated the impact of the p21 promoter-driven expression of TERT in obesity-induced metabolic disorders. The p21^+/Tert^ model in which the mCherry-2A-TERT cassette is inserted at the start codon of the p21^Cdkn1a^ gene has been described previously [22]. In addition, we generated here the p21^+/mCherry^ mouse as a control. These mice produce p21 protein from one allele and either mCherry alone or TERT and mCherry from the other allele (Figure 1A).

We fed p21^+/mCherry^ and p21^+/Tert^ male mice with standard chow diet (SD) as controls, or HFD for 8 weeks to induce obesity (Figure 1A). Body weight gain was significantly increased in HFD groups compared to SD fed male mice (Figure 1B) but we did not observe any difference in body weight gain between p21^+/−^ and p21^+/Tert^ obese mice (Figure 1B). Body weight gain was accompanied by increased accumulation of white AT (WAT) in all HFD mice, with no significant difference between groups (Figure 1C). Accordingly, we also observed an increase in the plasma leptin level, the main adipokine secreted by AT, in all groups of HFD mice with no difference between the genotypes (Figure 1D). Nevertheless, we observed that TERT expression counteracted both whole-body glucose intolerance and insulin resistance induced by HFD, as evidenced by a significant decrease in glycemia compared to HFD-fed p21^+/−^ male mice (Figure 1E–F). As expected, obese control mice exhibited a significant increase in plasma insulin compared to lean controls. In line with the enhanced insulin sensitivity observed in p21^+/Tert^ obese mice, p21-driven Tert expression restored plasma insulin levels to those seen in lean animals (Figure 1G).

Of note, no metabolic differences were observed between p21^+/mCherry^ and p21^+/Tert^ male mice under standard diet conditions (Fig. S1A–D). Similarly, no differences in metabolic profiles were observed between obese p21^+/mCherry^ and WT mice (Fig. S1E–I) indicating that monoallelic expression of p21 does not influence the metabolic outcomes.

We performed the same experiments on female mice (Fig. S2A). Similarly to male, body weight gain was significantly increased in HFD groups compared to SD fed mice (Fig. S2B) without any difference between p21^+/−^ and p21^+/Tert^ obese female mice (Fig. S2B). This was accompanied by increased accumulation of WAT and plasma leptin level in HFD mice, with no significant difference between groups (Fig. S2C–D). However, in female control mice (p21^+/−^), HFD induced significant but very mild glucose intolerance and insulin resistance (Fig. S2E–F). Therefore, we did not observe such improvement in p21^+/Tert^ obese female mice compared to p21^+/−^ obese female mice (Fig. S2E–F). Females are known to be more resistant against HFD induced metabolic disorders than male [23]. Notably, we found that the level of p21 in the AT of control females is much lower than that in control males on SD and it does not increase on HFD (Fig. S2G) that might explain the milder effect of TERT expression on glucose metabolism in females. In all the following experiments, only the data for male mice are presented.

Thus, expression of TERT under control of the p21 promoter leads to improved insulin resistance and reduced glucose intolerance in male mice fed a high-fat diet without any notable effect on the weight gain.

### 2.2 Reduced accumulation of p21 positive cells in the stromal vascular fraction (SVF) of AT

**Figure 2:**
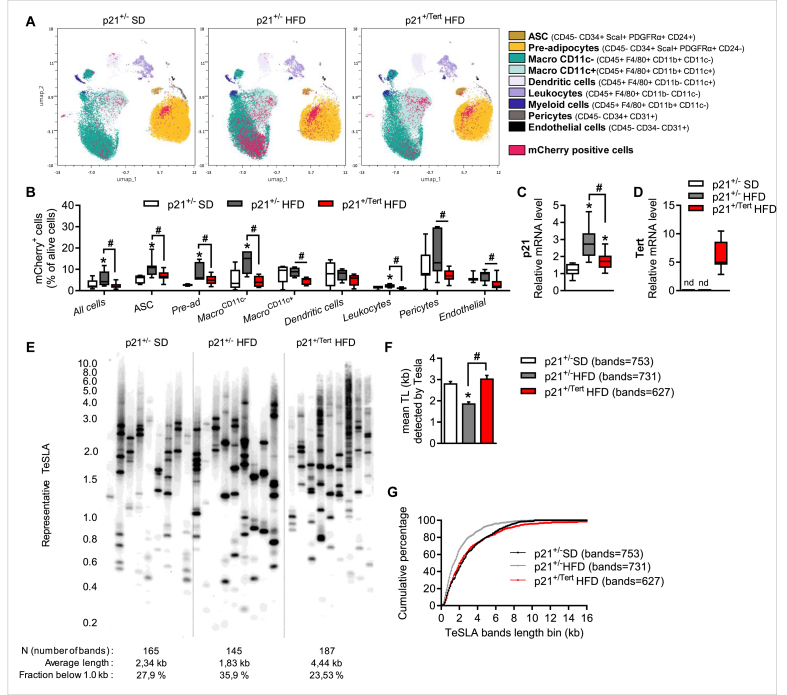
**Tert reduces the number of p21 positive cells in the stromal vascular fraction (SVF)** A. UMAP clustering of SVF cells. Cell populations were distinguished by FACS using the indicated markers (see Suppl Fig. 2). mCherry fluorescence was measured by FACS and signal quantification in all cells is indicated. (n = 6 mice per group) B. mCherry signal was measured by FACS. The percentage of mCherry positive cells is shown in the indicated populations of the SVF. (n = 5–8 p21^+/−^ SD, n = 4–7 p21^+/−^ HFD and n = 10–17 p21^+/Tert^ HFD and mice) C. p21 mRNA levels was measured by RT-qPCR in stromal vascular fraction (SVF) (n = 5 p21^+/−^ SD, n = 6 p21^+/−^ HFD and n = 6 p21^+/Tert^ HFD mice) D. Tert mRNA levels was measured by RT-qPCR in stromal vascular fraction (SVF) (n = 5 p21^+/−^ SD, n = 6 p21^+/−^ HFD and n = 6 p21^+/Tert^ HFD mice) E. Analysis of the short telomere fraction in the SVF cells isolated from the indicated mice by Telomere Shortest Length Assay (TeSLA). Genomic DNA was extracted from whole SVF; TeSLA was initiated with 50 ng of genomic DNA. In the final PCR step, 500 pg of ligation product was used per reaction, and 9 independent reactions were performed for each sample (each lane corresponds to an independent PCR reaction) to achieve >100 amplified telomeres for quantification. The panel depicts one representative Southern blot for the indicated mice probed for the TTAGGG repeats. (n = 3 per group). The percentage of bands less than 1 kb out of the total number of bands is indicated below the gel. F. Mean telomere length of the telomeres detected by the Tesla method. (n = 3 per group) G. Difference of cumulative number of short telomeres between SVF cells isolated from the indicated mice. (n = 3 per group). All data are shown as mean ± SEM. ∗p < 0.05 vs. p21^+/−^ SD mice (white bars), #p < 0.05 vs. p21^+/−^ HFD (grey bars). Student's t test or one-way ANOVA with Fisher multiple comparison test.

As p21 has been shown to play a crucial role in AT dysfunction in HFD-fed mice [16], and because TERT expression reduces p21 levels [22], we analyzed the number of mCherry-positive cells (reflecting p21 expression) in different cell types contained in the Stromal Vascular Fraction (SVF) namely pericytes, endothelial cells, ASPCs (adipose stem cells (ASC) and pre-adipocytes), leukocytes, monocytes, dendritic cells, macrophages that we defined at this stage as Macro CD11c^−^ and Macro CD11c^+^ [24]. The different cell subtypes were identified by FACS analysis using cell surface markers and precise gating strategy (Figure 2A and Fig. S4). This enabled us to group the different cell subtypes and identify the mCherry-expressing cells in each group, based on the fluorescence emitted by the latter (Figure 2A). We found that the mCherry signal was significantly increased by HFD in all cell types with a greater increase in ASCs, in pre-adipocytes and in macrophages (Figure 2A–B). We uncovered that the HFD-induced accumulation of mCherry-positive cells was reduced in p21^+/Tert^ obese mice in most of the SVF cell types (Figure 2A). Quantification of the fraction of mCherry positive cells in each cell type is shown in Figure 2B. Collectively, the results indicate that TERT expression significantly reduces the fraction of p21-positive cells. We further analyzed p21 expression by qPCR in the SVF (Figure 2C). We found that global p21 expression level was higher in obese compared to lean mice (Figure 2C). TERT expression significantly reduced p21 mRNA levels in mice fed HFD (Figure 2C). We then analyzed *Tert* expression by RT-qPCR in in the SVF of p21^+/−^ SD, p21^+/−^ HFD, and p21^+/Tert^ HFD mice. We found that TERT expression was barely detectable in p21^+/−^ SD and p21^+/−^ HFD mice, but increased significantly in p21^+/Tert^ HFD mice indicating that TERT expression is primarily derived from the transgene and not from the endogenous *Tert* gene (Figure 2D). This result is consistent with the fact that endogenous *TERT* is expressed only in a rare fraction of ASPCs [15]. Finally, we analyzed the telomere length of the SVF cells in the control SD-fed mice and in the HFD-fed mice of the 2 genotypes. Because mice have long telomeres making it difficult to detect variations in telomere length by classical Southern blots, and because short telomeres instigate tissues dysfunction, we evaluated the presence of short telomeres using TeSLA (TElomere Shortest Length Assay) [25]. This technique is specifically designed to detect a small fraction of all telomeres (typically shorter than ∼15 kb) by ligation-mediated PCR of individual chromosome ends. TeSLA allows the detection critically short dysfunctional telomeres that drive replicative senescence, thereby providing biologically meaningful insights into telomere dynamics in mouse models despite their long telomeres. The validity of TeSLA for the analysis of short telomeres in mice was addressed in previous studies [22,25]. To normalize the TeSLA, we use exactly the same amount of input DNA (see Methods) and calculate the total number of bands detected and the fraction of those smaller than 1 kb. As evident in the representative TeSLA (Figure 2E) and in the quantification graphs (Figure 2F–G), the obese control mice (p21^+/−^) had on average more short telomeres than lean mice suggesting that the HFD-induced stress may damage telomeres. In contrast, the cumulative percentage of short telomeres were similar between the lean p21^+/−^ and p21^+/Tert^ mice fed in HFD suggesting that p21 promoter-dependent expression of TERT heals the damaged telomeres (Figure 2F-G-H). Of note, we also detected HFD-induced telomere shortening and its attenuation by TERT in total AT but it was less pronounced compared to that in SVF (Fig. S5A–C). This may suggest that HFD affects telomere length primarily of the dividing cells in the SVF. Importantly, we analyzed the SVF (Fig. S3A–B), adipocyte size, distribution, and density (Fig. S3C-D-E-F), as well as the cellular composition of the SVF (Fig. S3G-H-I) in p21^+/−^ and p21^+/Tert^ mice maintained on a SD diet. No significant differences were found between the two groups except for a reduced number of mCherry-positive cells in the SVF of p21^+/Tert^ mice compared to p21^+/−^ controls. Similarly, the fraction of short telomeres did not differ between the two groups of mice fed an SD diet (Fig. S3J). These findings indicate that, in the absence of nutritional stress (i.e. HFD), young p21^+/Tert^ mice do not exhibit metabolic advantages over p21^+/−^ control mice.

### 2.3 TERT expression alleviates ASPC senescence induced by HFD

**Figure 3:**
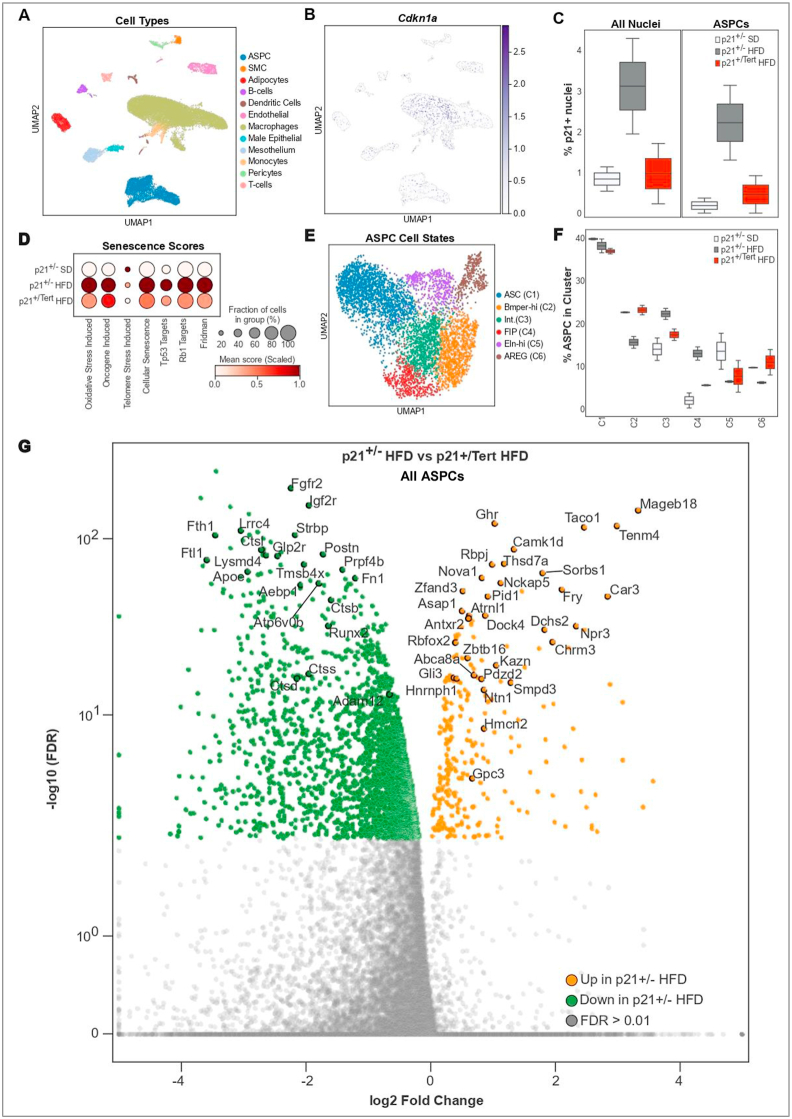
**Tert expression alleviates cellular senescence in ASPCs** A. UMAP of all cell populations with integration of p21^+/−^ SD, p21^+/−^ HFD and p21^+/Tert^ HFD mice B. UMAP of the p21 expression (Cdkn1a) in the different cell populations C. Proportion of p21+ cells across all cells and ASPCs in p21^+/−^ SD, p21^+/−^ HFD and p21^+/Tert^ HFD mice D. Senescence Scores in ASPCs in the different groups E. UMAP of ASPC cell stats F. Proportion of ASPC cell states in p21^+/−^ SD, p21^+/−^ HFD and p21^+/Tert^ HFD mice G. Volcano plot showing differential expression between combined ASPC subcluters of p21^+/−^ HFD and p21^+/Tert^ HFD mice. Orange represents upregulated genes, and green represents downregulated genes in p21^+/−^ HFD. Genes with an FDR>0.01 are colored in grey. Two-tailed Wilcoxon test with FDR correction. snRNA-seq was performed with two mice per group and detailed snRNA-seq methods are provided in the Supplemental Methods. (For interpretation of the references to color in this figure legend, the reader is referred to the Web version of this article.)

To assess the effect of TERT expression controlled by the p21 promoter in specific AT cell types and especially ASPCs, we performed single-nucleus RNA sequencing (snRNA-seq, Supplemental Methods) on WAT from p21^+/−^ mice fed with SD and p21^+/−^ and p21^+/Tert^ fed with HFD for 8 weeks. After quality filtering, we generated dataset for p21^+/−^ SD (n = 2), p21^+/−^ HFD (n = 2), and p21^+/Tert^ HFD (n = 2). We integrated the datasets and performed dimensionality reduction and unsupervised cell clustering to identify distinct cell types based on unique and shared gene expression patterns [26,27] (Figure 3A and Fig. S6B). The number of cells for each cell type and for each condition is indicated in the Dataset 1. For all mice, we recovered nuclei from the main cell types reported to be present in the WAT [16,26]. We analyzed p21 expression in the different AT cell types in p21^+/−^ and p21^+/Tert^ mice fed with HFD (Figure 3B). In HFD-fed control mice (p21^+/−^), p21 expression was mainly induced in ASPCs and macrophages (Figure 3B). In agreement with our earlier observations in SVF (Figure 2), the fraction of p21-positive cells among all AT cell types was markedly elevated in HFD-fed mice (p21^+/−^), and reduced in p21^+/Tert^ HFD mice (Figure 3C).

The fact that the number of p21-positive cells was markedly reduced in ASPCs of p21^+/Tert^ HFD mice prompted us to assess senescence signatures in the different mice fed with HFD. We revealed increased expression of the senescence-associated genes, notably targets of Tp53 and Rb in the ASPCs of p21^+/−^ mice exposed to HFD. Strikingly, up-regulation of the senescence gene scores was strongly decreased in ASPCs from p21^+/Tert^ mice (Figure 3D). In addition, the increase in senescence associated with HFD is also diminished in other cell types derived from the fat of p21^+/Tert^ mice such as adipocytes and macrophages (Fig. S6) and Fig. S6B). Overall, these results suggest that p21-promoter dependent expression of *Tert* alleviates the accumulation of senescent cells in ASPCs and other cell types in obese mice.

Since adipose stem and progenitor cells are critical in AT homeostasis, we next used unsupervised clustering and marker gene expression to divide ASPC into subtypes (Figure 3E). This revealed a cluster of early progenitors (ASC expressing CD55 and DDP4) and 5 more committed progenitor clusters that shared transcriptional signatures with previously reported clusters (Fig. S6C) [8,26,[28], [29], [30]]. Of the committed progenitor clusters, C6 was consistent with adipogenesis regulatory (AREGs) precursors [26,28,30], C2 expressed *Bmper* which has recently been shown to have pro-adipogenic functions [30], and C4 expressed pro-inflammatory and pro-fibrotic genes as well as *Notch3* which has reported anti-adipogenic effects (SF6A) [28,29]. Differential abundance analyses in ASPC subpopulations supported a relative increase in fibroinflammatory (C4) precursors in HFD compared to SD, and a relative reduction in pro-adipogenic C2 cells overexpressing *Bmper* (Figure 3F). This pattern was reversed in p21^+/Tert^ HFD mice, which had reduced FIP and increased BMPER proportions compared to HFD, suggesting HFD may shift ASPC towards more adverse subtypes and that p21^+/Tert^ may attenuate this effect (Figure 3F).

We then identified differentially expressed genes (DEGs) across the entire ASPC cluster from p21^+/−^ and p21^+/Tert^ mice fed HFD (Figure 3G) to obtain a comprehensive overview of how Tert expression may influence ASPCs function and differentiation. The down-regulated genes between ASPCs from p21^+/−^ and p21^+/Tert^ obese mice include Ftl1, Fth1, Ctsl, Postn, Fgfr2, Igf2r while upregulated genes include Ghr, Zbtb16, Nova1, and Gli3 (Dataset 2) (see discussion). Interestingly, it was very recently reported that the specific inactivation of Fth1 in AT is accompanied by a decrease in mitochondrial ROS levels, increased insulin sensitivity and glucose tolerance [31]. Nova1 attracted our interest as a splicing factor that interacts with *hTERT* pre-mRNA, facilitating the production of full-length, active hTERT [32]. It has also been reported to promote the expression of adipogenic genes in mesenchymal stem cells (MSCs) from white adipose tissue [33]. We also found that genes described as negative regulators of the Wnt/β-catenin signaling pathway were overexpressed in p21^+/Tert^ ASPC (Dataset 2). This is illustrated by the up-regulation of Ghr, Gli3, and Gpc3, that have been reported to promote adipogenesis by counteracting activation of the Wnt pathway (see discussion) [[34], [35], [36]].

Together, snRNA-seq results confirmed that HFD promotes senescence in AT and ASPCs, while p21^+/Tert^ under HFD reduces it, and revealed ASPC subtype and gene expression differences linked to tissue changes.

### 2.4 Tert inhibits ASPC senescence *in vitro* and improves their differentiation

**Figure 4:**
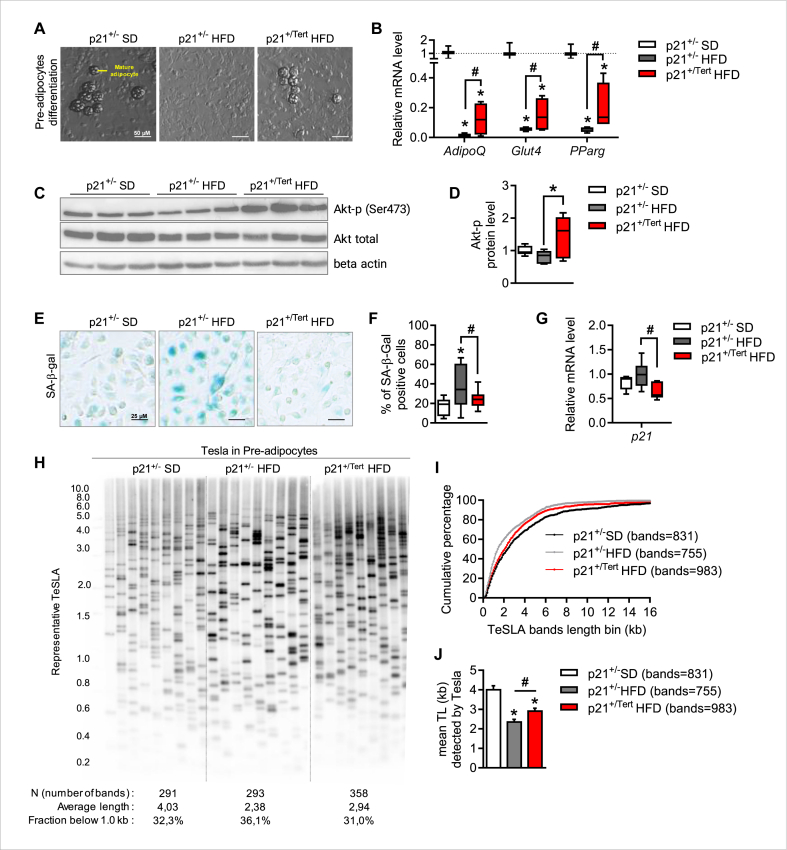
**ASPCs isolated from SVF of HFD p21^+/Tert^ mice show reduced senescence and enhanced differentiation** A. Representative photographs showing adipocytes formation from ASPCs in cell culture. Scale is indicated on the image. B. Expression of the genes involved in lipogenesis was measured by RT-qPCR in ASPCs cell cultures established from WAT of the p21^+/−^ SD, p21^+/−^ HFD and p21^+/Tert^ HFD mice (n = 3 p21^+/−^ SD, n = 4 p21^+/−^ HFD and n = 4 p21^+/Tert^ HFD mice) C. Representative Western blot of phosphorylated Akt protein level in ASPCs established from WAT of the p21^+/−^ SD, p21^+/−^ HFD and p21^+/Tert^ HFD mice D. Quantification of phosphorylated Akt protein level (n = 6 p21^+/−^ SD, n = 5 p21^+/−^ HFD and n = 5 p21^+/Tert^ HFD mice) E. SA-β-gal staining of cultured ASPCs from the WAT of the p21^+/−^ SD, p21^+/−^ HFD and p21^+/Tert^ HFD mice. Scale is indicated on the image. (n = 3 p21^+/−^ SD, n = 4 p21^+/−^ HFD and n = 4 p21^+/Tert^ HFD mice). F. Quantification of SA-β-gal positive cells in the cultures established from WAT of the indicated groups of mice (n = 3 p21^+/−^ SD, n = 4 p21^+/−^ HFD and n = 4 p21^+/Tert^ HFD mice) G. p21 mRNA expression was measured by RT-qPCR in ASPC cell cultures established from WAT of the p21^+/−^ SD, p21^+/−^ HFD and p21^+/Tert^ HFD mice (n = 5 p21^+/−^ SD, n = 7 p21^+/−^ HFD and n = 7 p21^+/Tert^ HFD mice) H. The shortest telomere burden in ASPCs analyzed by TeSLA (See Figure 2E for details) (n = 3 per group) I. Difference between the cumulative numbers of short telomeres for the cultured ASPCs isolated from the SVF of the indicated mice. Three independent cultures were analyzed for the indicated mice. (n = 3 per group) J. Mean telomere length of the telomeres detected by the TeSLA method in the indicated groups of mice (n = 3 per group). All data are shown as the mean ± SEM. ∗p < 0.05 vs. p21^+/−^ SD mice (white bars), #p < 0.05 vs. p21^+/−^ HFD (grey bars). Student's t test or one-way ANOVA with Fisher multiple comparison test.

We aimed at consolidating the above results by culturing ASPC isolated from the 3 different groups of mice. Briefly, WAT was collected from SD and HFD p21^+/−^ mice and from HFD p21^+/Tert^ mice and primary ASPCs were isolated and cultured accordingly to established protocol [37]. At confluence, the differentiation of ASPCs into mature adipocytes was stimulated by medium containing insulin. We observed that differentiation was reduced in p21^+/−^ obese mice compared to ASPCs isolated from SD-fed p21^+/−^ mice (Figure 4A). This differentiation defect was attenuated in ASPCs from p21^+/Tert^ obese mice (Figure 4A). Additionally, expression of the adipogenic genes (adiponectin, Glut4 and Pparγ) was significantly enhanced in p21^+/Tert^ ASPCs compared to p21^+/−^ASPCs (Figure 4B), confirming improved differentiation of ASPCs into mature adipocytes in p21^+/Tert^ obese mice compared with obese p21^+/−^ mice. Consistent with these results, we observed a significant increase in phosphorylated Akt levels in ASPCs from p21^+/Tert^ obese mice compared to p21^+/−^ obese controls, indicating enhanced insulin sensitivity (Figure 4C–D).

We then performed senescence-associated β-galactosidase (SA-βGal) staining of ASPCs to assess senescence induction. In agreement with previous studies [19,38], we observed a greater number of SA-βGal positive cells in ASPCs from obese p21^+/−^ mice compared with ASPCs from p21^+/−^ mice fed in SD, reflecting an increase in senescence caused by the HFD [39] (Figure 4E–F). In contrast, ASPCs derived from p21^+/Tert^ obese mice did not show elevated levels of senescence (Figure 4E–F). In line with our previous results, p21^+/−^ HFD ASPCs showed higher p21 expression than those from SD-fed mice (Figure 4G). We again observed a lower percentage of ASPCs positive for p21 in p21^+/Tert^ mice when compared to that in p21^+/−^ mice (Figure 4G). We wondered whether the senescence observed in the ASPCs of obese p21^+/−^ mice correlated with telomere shortening in these mice. We thus analyzed short telomeres by TeSLA [25] in the cultured ASPCs from SD and HFD mice of the 3 genotypes. We observed that ASPCs from obese p21^+/−^ mice had shorter telomeres than ASPCs from lean mice (Figure 4H-I-J). Similar to what we observed in SVF (Figure 2D), HFD-induced telomere shortening was counteracted by TERT expression in ASPCs from obese p21^+/Tert^ mice (Figure 4H-I-J). We infer from the TeSLA results that p21^+/−^ASPCs senescence might be driven by increased telomeric damage [40].

### 2.5 Tert expression improves ASPC's mitochondrial function and reduces oxidative stress

**Figure 5:**
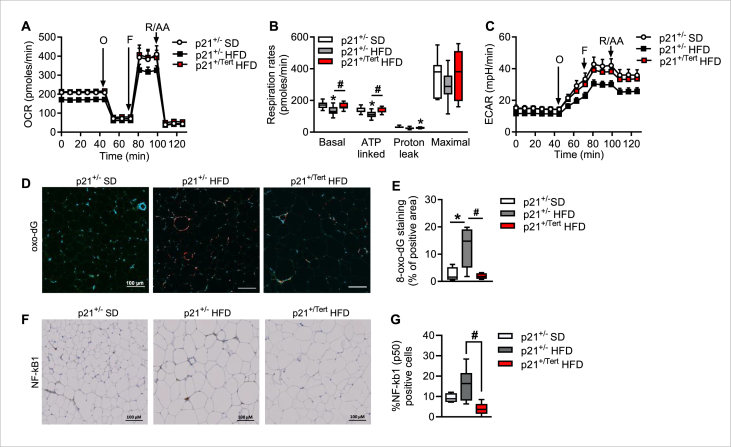
**Tert expression reduces oxidative stress and mitochondrial dysfunction induced by obesity** A. Oxygen consumption rate analyzed by Seahorse in ASPC cell cultures established from WAT of the p21^+/−^ SD, p21^+/−^ HFD and p21^+/Tert^ HFD mice (n = 3 p21^+/−^ SD, n = 4 p21^+/−^ HFD and n = 4 p21^+/Tert^ HFD mice in duplicate) B. Respiratory rates calculated based on oxygen consumption data (n = 3 p21^+/−^ SD, n = 4 p21^+/−^ HFD and n = 4 p21^+/Tert^ HFD mice in duplicate) C. Extracellular acidification rate (ECAR), an index of glycolysis (n = 3 p21^+/−^ SD, n = 4 p21^+/−^ HFD and n = 4 p21^+/Tert^ HFD mice in duplicate) D. 8-Oxo-désoxyguanosine (oxo-dG) staining of visceral adipose tissue (WAT) from p21^+/−^ SD, p21^+/−^ HFD and p21^+/Tert^ HFD mice. (n = 3 p21^+/−^ SD, n = 4 p21^+/−^ HFD and n = 4 p21^+/Tert^ HFD mice) E. Quantification of the oxo-dG positive cell (n = 3 p21^+/−^ SD, n = 4 p21^+/−^ HFD and n = 4 p21^+/Tert^ HFD mice) F. NF-kB (p50) staining of visceral adipose tissue (WAT) from p21^+/−^ SD, p21^+/−^ HFD, p21^+/Tert^ mice. (n = 4 p21^+/−^ SD, n = 9 p21^+/−^ HFD, n = 7 p21^+/Tert^ HFD HFD) G. Quantification of the NF- kB (p50) positive cell (n = 4 p21^+/−^ SD, n = 9 p21^+/−^ HFD, n = 7 p21^+/Tert^ HFD). All data are shown as the mean ± SEM. ∗p < 0.05 vs. p21^+/−^ SD mice (white bars), #p < 0.05 vs. p21^+/−^ HFD (grey bars). Student's t test or one-way ANOVA with Fisher multiple comparison test.

Telomere dysfunction in mice has been associated with impaired mitochondrial function and increased reactive oxygen species [41,42]. Moreover, in cell models, TERT has been shown to localized in mitochondria upon oxidative stress and to decrease ROS generation [43,44]. These studies combined to the above results prompted us to assess the impact of p21 promoter-driven expression of TERT on the bioenergetics of the cultured ASPCs. Using the Seahorse analyzer, which measures the rate of oxygen consumption in the cell culture medium, reflecting the mitochondrial function of the cells, we found that ASPCs isolated from p21^+/−^ obese mice showed a slight decrease in basal and ATP-related respiration compared with ASPCs isolated from SD p21^+/−^ mice (Figure 5A–B) indicating that HFD treatment induces mitochondrial dysfunction in ASPCs. Strikingly, we observed that p21^+/Tert^ ASPCs displayed similar respiratory rates than ASPCs isolated from p21^+/−^ mice fed in SD (Figure 5A–B) suggesting that that conditional expression of TERT preserves mitochondrial function in mice fed with HFD. Of note, we also observed that glycolysis was also preserved in p21^+/Tert^ ASPCs compared to p21^+/−^ASPCs from obese mice (Figure 5C).

We have previously shown that the overall level of oxidative damage was reduced in the lungs of aged p21^+/Tert^ mice [22]. We therefore wondered whether this was also the case in the AT of p21^+/Tert^ mice fed with HFD. We analyzed the overall level of oxidative DNA damage by measuring 8-oxo-dG levels in the AT of p21^+/−^ and p21^+/Tert^ mice fed with HFD. We found that 8-oxo-dG levels were increased in the AT of HFD-fed p21^+/−^ mice, but that this increase in oxidative damage was abolished in obese p21^+/Tert^ mice (Figure 5D–E).

As an additional marker of senescence, we assessed the expression of the p50 NF-kB subunit (encoded by Nfkb1). NF-κB is a key regulator of the SASP, and the expression of different NF-κB subunits is upregulated in senescent cells [45,46]. We and others have previously measured the expression of NF-κB subunits as a surrogate of the SASP [47]. Notably, we observed a reduction in p50 NF-κB foci in p21^+/Tert^ mice fed a high-fat diet (HFD) compared to p21^+/−^ HFD controls, supporting the conclusion that Tert expression mitigates cellular senescence in adipose tissue under obesogenic conditions (Figure 5F–G).

In summary, expression of TERT under the control of the p21-promoter strongly reduces DNA oxidative damage, senescence markers and improves mitochondrial function and glycolysis in ASPCs cultured *in vitro* harvested from the AT of obese mice.

#### 2.6 Formation of mature adipocytes is improved in p21^+/Tert^ obese mice

**Figure 6:**
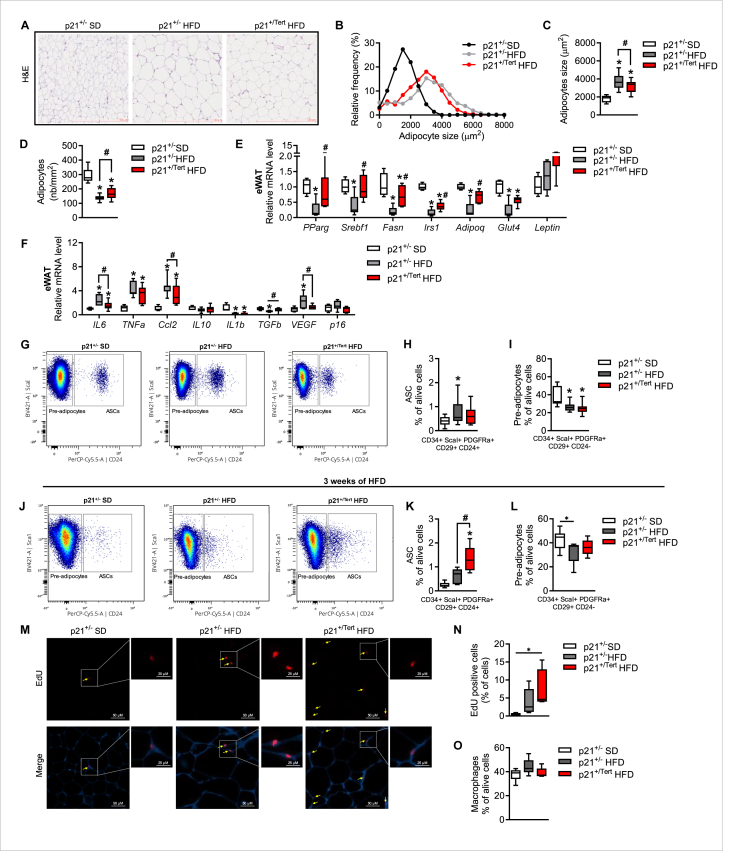
**Tert improves formation of adipocytes in obese mice** A. Hematoxylin and eosin (H&E) staining of visceral adipose tissue (WAT) from p21^+/−^ SD, p21^+/−^ HFD and p21^+/Tert^ HFD mice. B. Relative frequency of adipocyte sizes in the indicated groups of mice was plotted using GraphPad (n = 5 p21^+/−^ SD, n = 13 p21^+/−^ HFD and n = 15 p21^+/Tert^ HFD mice) C. Adipocytes size in the images of H&E-stained WAT was quantified using AdipoSoft ImageJ software (n = 5 p21^+/−^ SD, n = 13 p21^+/−^ HFD and n = 15 p21^+/Tert^ HFD mice) D. Adipocyte cell density in the images of H&E-stained WAT was quantified using AdipoSoft ImageJ software (n = 5 p21^+/−^ SD, n = 13 p21^+/−^ HFD and n = 15 p21^+/Tert^ HFD mice) E. Expression of the genes involved in lipogenesis, lipolysis and fatty acid oxidation was measured by RT-qPCR in eWAT of the p21^+/−^ SD, p21^+/−^ HFD and p21^+/Tert^ HFD mice (n = 5 p21^+/−^ SD, n = 6 p21^+/−^ HFD and n = 6 p21^+/Tert^ HFD mice) F. Expression of the genes involved in inflammation was measured by RT-qPCR in eWAT of the p21^+/−^ SD, p21^+/−^ HFD and p21^+/Tert^ HFD mice (n = 5 p21^+/−^ SD, n = 6 p21^+/−^ HFD and n = 6 p21^+/Tert^ HFD mice) G. Representative flow cytometry data of ASPCs (pre-adipocytes and ASCs) after 8 weeks of HFD H. Relative percentage of Adipose Stem and Progenitors Cells (ASPC) in the WAT of the p21^+/−^ SD, p21^+/−^ HFD and p21^+/Tert^ HFD mice analyzed by FACS (n = 13 p21^+/−^ SD, n = 11 p21^+/−^ HFD and n = 15 p21^+/Tert^ HFD mice) I. Relative percentage of pre-adipocytes in the WAT of the p21^+/−^ SD, p21^+/−^ HFD and p21^+/Tert^ HFD mice analyzed by FACS (n = 13 p21^+/−^ SD, n = 11 p21^+/−^ HFD and n = 15 p21^+/Tert^ HFD mice) J. Representative flow cytometry data of ASPCs (pre-adipocytes and ASCs) after 3 weeks of HFD K. Relative percentage of Adipose Stem and Progenitors Cells (ASPC) in the WAT of the p21^+/−^ SD, p21^+/−^ HFD and p21^+/Tert^ HFD mice analyzed by FACS (n = 6 p21^+/−^ SD, n = 6 p21^+/−^ HFD and n = 7 p21^+/Tert^ HFD mice) L. Relative percentage of pre-adipocytes in the WAT of the p21^+/−^ SD, p21^+/−^ HFD and p21^+/Tert^ HFD mice analyzed by FACS (n = 6 p21^+/−^ SD, n = 6 p21^+/−^ HFD and n = 7 p21^+/Tert^ HFD mice) M. EdU staining into APSC from male WAT from p21^+/−^ SD, p21^+/−^ HFD and p21^+/Tert^ HFD mice after 48 h pulses of EdU (n = 4 p21^+/−^ SD, n = 5 p21^+/−^ HFD and n = 4 p21^+/Tert^ HFD mice) N. Quantification of EdU positive cells (n = 4 p21^+/−^ SD, n = 5 p21^+/−^ HFD and n = 4 p21^+/Tert^ HFD mice) O. Relative percentage of Macrophages in the WAT of the p21^+/−^ SD, p21^+/−^ HFD and p21^+/Tert^ HFD mice analyzed by FACS (n = 6 p21^+/−^ SD, n = 5 p21^+/−^ HFD and n = 6 p21^+/Tert^ HFD mice). All data are shown as the mean ± SEM. ∗p < 0.05 vs. p21^+/−^ SD mice (white bars), ^#^p < 0.05 vs. p21^+/−^ HFD (grey bars). Student's t-test or one-way ANOVA with Fisher multiple comparison test.

To further confirm the effect of TERT on adipocyte formation, we performed a histological analysis of the AT of the HFD mice. We first noticed that, despite no difference in WAT weight between obese mice (Figure 1C), the adipocyte size in the WAT of obese p21^+/Tert^ mice was reduced, with smaller and denser adipocytes compared to obese control mice (Figure 6A). We found that HFD induced a significant increase in adipocyte size in p21^+/−^ obese mice, evidenced by a shift toward a higher proportion of large adipocytes in the adipocyte size distribution (Figure 6B), an increased in mean adipocyte size (Figure 6C), and a reduction in adipocyte density (Figure 6D). In contrast, p21^+/Tert^ obese mice displayed smaller adipocytes, as indicated by a shift toward fewer large adipocytes (Figure 6B), a lower mean adipocyte size (Figure 6C), and increased adipocyte density (Figure 6D), relative to p21^+/−^ obese mice.

In addition, we found that expression (analyzed by qPCR) of a number of adipocyte markers [adiponectin (AdipoQ), Glucose transporter 4 (Glut4), peroxisome proliferator-activated receptor gamma (PPARγ), fatty acid synthase (Fasn), sterol regulatory element-binding transcription factor 1 (Srebp1c), and Insulin receptor 1 (Irs1)] was down-regulated in p21^+/−^ fed with HFD, whereas it was partially maintained in p21^+/Tert^ HFD mice (Figure 6E). Consistent with previous reports showing that early HFD-induced progenitor proliferation is restricted to visceral, but not subcutaneous, adipose tissue [48], we found that adipogenic gene expression was similarly not restored in the subcutaneous (SubCut WAT) of p21+/Tert obese mice (Fig. S7).

We also examined inflammation-related genes (IL-6, TNFα, Ccl2, IL-10, IL-1b, Tgf-beta, VEGF, and p16) and found that the improvement in adipose tissue function was accompanied by a less inflammatory profile, as indicated by reduced expression of IL6, TNFα, Ccl2, and VEGF (Figure 6F).

FACS analysis of the WAT revealed an accumulation of ASPCs in the SVF of p21^+/−^ obese mice while the number of ASPCs in obese p21^+/Tert^ mice was at the same level as in SD-fed control mice (Figure 6G–H). Conversely, the number of pre-adipocytes was similar in the p21^+/−^ and p21^+/Tert^ obese mice (Figure 6G–I). Collectively, these results suggest that mature adipocyte formation is improved in p21^+/Tert^ obese mice.

To determine whether TERT stimulates ASPC proliferation in the WAT of p21^+/Tert^ mice fed with HFD, we fed the mice with HFD over a shorter period (3 weeks instead of 8). Indeed, since ASC proliferation peaks between 1 and 3 weeks of HFD [[48], [49], [50]] and p21 expression rises between 2 and 5 weeks [19], we selected the 3-week time point to capture both the transient ASC proliferation and the onset of p21-driven Tert expression. This timing allowed us to specifically assess the effect of p21-mediated Tert activation.

ASC form a pool of stem cells with adipogenic potential that become further committed to the adipocyte lineage as they lose CD24 expression, forming a CD24 negative pre-adipocyte population in the course of differentiating into mature adipocytes [5,6]. In the early response to the diet (3 weeks), FACS analysis reveal a higher number of ASCs (CD29^+^;CD34^+^;Sca1^+^;CD24^+^) (Figure 6J–K) and pre-adipocytes (CD29^+^;CD34^+^;Sca-1^+^;CD24^-^) (Figure 6J-L) in the SVF of p21^+/Tert^ obese mice compared with p21^+/−^ obese mice. This suggests that Tert overexpression promotes expansion and differentiation of ASCs into pre-adipocytes at early stage of HFD. This then leads to higher number of mature adipocytes at 8 weeks of HFD (Figure 6A–D) as well as increase expression of adipogenic genes (Figure 6E) in the WAT of p21^+/Tert^ obese mice compared to control obese ones.

To further explore this observation, mice were injected intraperitoneally with clickable EdU after 3 weeks of HFD and 48 h before sacrifice. EdU incorporation was revealed by click-it staining on paraffin sections, and EdU-positive cells were quantified in adipose tissue. We observed an increase, of about 2-fold, in the number of EdU-positive cells in p21+/Tert obese mice compared with SD controls (Figure 6M−N).

In parallel, we assessed macrophages (CD45^+^; F4/80^+^; CD11b^+^; CD11c^+^) by FACS after 3 weeks. p21^+/−^ obese mice showed a slight, but not significant, increase in macrophage numbers, whereas p21^+/Tert^ obese mice displayed a modest decrease compared to p21^+/−^ obese mice (Figure 6O).

Although EdU incorporation reflects overall proliferative activity within adipose tissue, the lack of macrophage expansion combined with the increase in ASPCs by FACS suggest that the higher EdU signal may largely derive from enhanced ASC proliferation *in vivo* following Tert expression.

### 2.7 Improvement of AT function diminishes hepatic steatosis induced by HFD in p21^+/Tert^ mice

**Figure 7:**
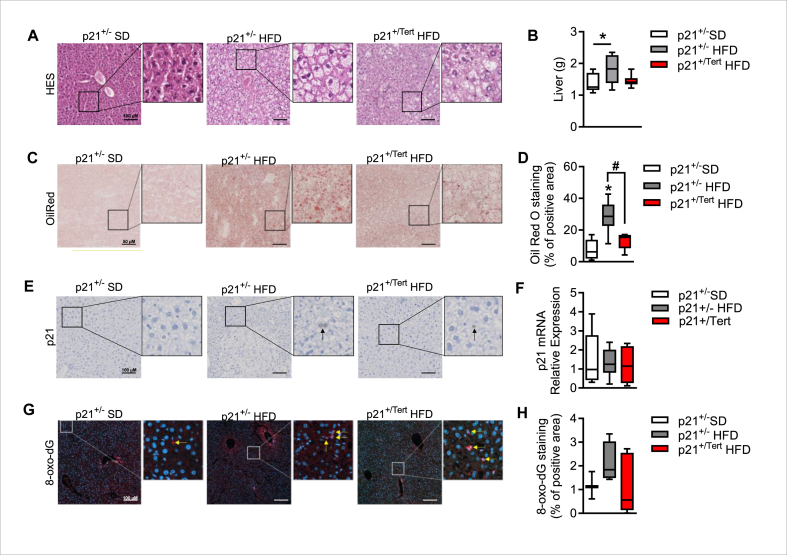
**Improvement of adipose tissue function diminishes hepatic steatosis in p21^Tert^ obese mice** A. Hematoxylin and eosin (H&E) staining of Liver from p21^+/−^ SD, p21^+/−^ HFD and p21^+/Tert^ HFD mice. B. Liver weight (g) at sacrifice (n = 6 p21^+/−^ SD, n = 7 p21^+/−^ HFD and n = 7 p21^+/Tert^ HFD mice) C. Oil red O staining of Liver from p21^+/−^ SD, p21^+/−^ HFD and p21^+/Tert^ HFD mice. D. Quantification of OilRed O staining (n = 5 p21^+/−^ SD, n = 6 p21^+/−^ HFD and n = 6 p21^+/Tert^ HFD mice) E. p21 staining of Liver from p21^+/−^ SD, p21^+/−^ HFD and p21^+/Tert^ HFD mice. F. mRNA relative expression of p21 of Liver from p21^+/−^ SD, p21^+/−^ HFD and p21^+/Tert^ HFD mice measured by qPCR (n = 5 p21^+/−^ SD, n = 6 p21^+/−^ HFD and n = 6 p21^+/Tert^ HFD mice). G. 8-Oxo-desoxyguanosine (oxo-dG) staining of liver H. Quantification of oxo-dG staining (n = 5 p21^+/−^ SD, n = 6 p21^+/−^ HFD and n = 6 p21^+/Tert^ HFD mice). All data are shown as the mean ± SEM. ∗p < 0.05 vs. p21^+/−^ SD mice (white bars), ^#^p < 0.05 vs. p21^+/−^ HFD (grey bars). One-way ANOVA with Fisher multiple comparison test. (For interpretation of the references to color in this figure legend, the reader is referred to the Web version of this article.)

In our mouse model, TERT expression under the control of the p21 promoter is not limited to AT and other organs may be involved in the beneficial effects conferred by TERT expression. Therefore, we analyzed the liver, another important metabolic organ. We observed that the increased liver volume and hepatic steatosis induced by HFD in p21^+/−^ control mice was reduced in p21^+/Tert^ mice (Figure 7A–D). However, unlike AT, only 2 months of HFD diet was not sufficient to increase the number of p21-positive cells in the liver of obese mice (Figure 7E–F), nor to cause an increase in 8-oxodG damage (Figure 7G–H). These results support the notion that AT is the organ primarily involved in the beneficial effects observed in p21^+/Tert^ mice fed with HFD.

## 3 Discussion

Obesity is regarded as a disease of excess AT leading to metabolic disorders [51]. However, risk of metabolic dysfunction in obesity appears to be determined by limited capacity for AT remodelling [52,53]. Telomere shortening-induced senescence has emerged as an important contributor to AT remodeling dysfunction during obesity [54]. Indeed, senescent adipocyte progenitors lose their self-renewal capacity and differentiation potential, contributing to hypertrophic adipocytes formation and metabolic complications in mice and humans [8,13,55]. Cellular senescence, in addition to being a player in AT dysfunction [52], is above all a hallmark of aging [56,57], conferring the status of “aging-like” pathology to obesity.

Here, we report that adipose cell progenitors from obese mice display telomere shortening, high p21 expression, increased SA-β-galactosidase activity, decreased adipogenic and differentiation potentials, and mitochondrial dysfunction. We show that the p21 promoter-dependent expression of TERT in HFD fed mice is associated with reduced p21 expression in particular in ASPCs, prevention of telomere attrition, decrease of DNA oxidative damages, and reduced senescence. This facilitates expansion and differentiation of stem and progenitor cells into mature adipocytes as shown by *in vivo* and *in vitro* experiments. These results are consistent with previous findings that mice with a double deletion of the p21/p27 genes develop obesity due to increased adipocyte hyperplasia [58]. We propose that all these changes, in particular enhanced adipocyte formation, play an important role in improving insulin-resistance and reducing glucose intolerance in p21^+/Tert^ mice fed on a HFD.

In obesity, expansion of the AT is driven by an increase in number and in size of adipocytes. From a metabolic point of view, adipocyte hyperplasia is more favorable than adipocyte enlargement [59]. We show that after 8 weeks on HFD, the AT from obese mice expressing TERT contains a greater number of adipocytes and of smaller in size compared to control mice. Remarkably, we show that after 3 weeks in HFD, the number of dividing cells within the AT is 2 times greater in p21^+/Tert^ mice than in p21^+/−^ mice. Moreover, *in vivo* and *in vitro* experiments indicate formation of functional adipocytes is promoted by TERT. In cultured ASPCs from obese mice, p21 promoter-driven expression of TERT reduces senescence and alleviates mitochondrial dysfunction. Of note, in our *in vitro* experiments, ASPCs were enriched by plating the SVF and selecting for adherent cells rather than by cell sorting, which may have resulted in a partially heterogeneous cell population and thus represents a limitation of our study. Nevertheless, Tert expression in this context likely creates a supportive microenvironment that promotes adipose progenitor differentiation.

Numerous studies have shown that TERT protects against oxidative stress, a function linked to its mitochondrial localization and the associated reduction in reactive oxygen species levels [43,60,61]. Using mouse models expressing TERT exclusively in either the mitochondria or the nucleus, it was demonstrated that mitochondrial TERT confers cardio-protection, in particular by enhancing mitochondrial complex I activity [62]. In this study, we demonstrated that levels of 8-oxo-dG and Nfkb1 were reduced in the AT of p21^+/Tert^ mice fed a high-fat diet (HFD). Additionally, *in vitro* conditional expression of TERT in ASPCs isolated from obese mice preserved mitochondrial function. Whether the observed reduction in oxidative damage, decreased Nfkb1 levels, and TERT-enhanced mitochondrial activity are mechanistically linked remains to be determined. Further studies are warranted to clarify whether telomere length contributes to the mitochondrial effects observed in this study as previous reports have shown that TERT loss can affect mitochondrial function independently of telomere length [63].

Of note, in a previous study [64], a transient induction of *TERT* expression and telomerase activity was detected during pre-senescence stage of the fibroblasts with shortened telomeres obtained from the progenies of late-generation *Tert*^+/−^ breeding. Transient activity of telomerase observed during pre-senescence of the *Tert*^+/+^ fibroblasts altered the dynamics of DNA Damage Response and delayed senescence relative to the *Tert*^−/−^counterparts [64].

We propose that in HFD-fed mice, TERT by inhibiting p21 expression and senescence pathways facilitates the exit of adipose stem cells from quiescence [65] and promote their further expansion. Proliferation of ASPCs may then lead to an accumulation of very short telomeres, probably due to replicative stress, that will be repaired by catalytically active TERT. At a later stage, TERT maintains a healthy pool of adipocyte precursors which are committed to differentiation into adipocytes. Our results are consistent with a recent study from Wang and colleagues that showed that specific elimination of p21^high^ cells prevents insulin resistance in obese mice. Similar to our observations, they reported that p21^high^ cells in AT were mainly ASPCs and macrophages [16].

Changes in the expression of a number of genes in the ASPC of the AT mice could explain the specific features of the p21^+/Tert^ ASPC. For instance, we found that Fgfr2 that is known to be a driver of osteoblast differentiation and expressed in adipocytes [66,67] is down regulated in p21^+/Tert^ ASPCs. Other genes playing a role in bone formation (Postn, Aebp1, Adam12, Runx2) as well as a number of cathepsin and related proteins (Ctsl, Ctsb, Ctsd, Ctsp, Ctss) that promote osteocyte differentiation [68] were found also to be down regulated (Dataset 2). These changes are anticipated to promote ASPCs commitment to adipogenic differentiation. Along the same line, we found that the conditional expression of TERT promotes the up-regulation of a number of genes (Ghr/Gli3/Gpc3) (Dataset 2) described as negative regulators of the canonical Wnt/β-catenin signaling pathway. Because Wnt signaling preserves progenitor cell multipotency during the early stage of adipogenesis while its subsequent inactivation is necessary for ASPSc differentiation into adipocytes [69,70], it is conceivable that TERT promotes ASPCs commitment to the adipocyte differentiation by reducing Wnt signaling. Of note, the multifaceted transcription factor Zbtb16 (PLZF) [71] was overexpressed in ASPCs from p21^+/Tert^ HFD mice. Zbtb16 draws our attention because it has been reported to directly repress the transcription of *Cdkn1a* by binding to proximal Sp1-binding GC-box 5/6 and distal Tp53-responsive elements of the p21 promoter [72]. Another important piece of information lies in the fact that expression of p21 and senescence-associated genes is reduced in ASPCs from the AT of p21^+/Tert^ HFD mice. Lower p21 level in p21^+/Tert^ HFD obese mice is expected to promote the activity of Cdk2 and Cdk4 that has been reported to favor clonal expansion and differentiation of adipocyte precursors [73].

Another potential mechanism underlying the metabolic benefits is the modulation of adipose tissue inflammation. The qPCR data show a reduction in pro-inflammatory markers in p21^+/Tert^ obese mice. Therefore, in addition to promoting adipogenesis, the decrease in stromal cell senescence may contribute to improve insulin sensitivity by lowering inflammatory signaling and restoring healthier endocrine–paracrine communication between adipocytes and immune cells.

Importantly, our results indicate that it is mainly males that are metabolically affected by HFD in the short-term (here 8 weeks), while females are protected against HFD-induced insulin resistance and glucose intolerance [23]. Many studies have attributed this protection to estrogen, since older post-menopausal female mice are no longer protected against obesity [74,75]. In our study, obese females did not show higher expression of p21 in AT than lean females, suggesting that female mice are better protected in particular against HFD-induced p21-expression in the AT. Whether this is due to a better protection to DNA damage remains to be determined. Gao and colleagues also found that clearance of p21^high^ cells had less metabolic benefits in female than male obese mice after 2 months HFD [13]. These results support the idea that female, somehow, are better protected against senescence and may therefore explain sex-differences in senescence-associated diseases. It would be therefore of great interest to extend these investigations in female mice to determine whether p21-driven Tert expression similarly affects mechanisms observed in males, such as telomere length, oxidative damage, and related pathways.

Taken together, these results support the idea that the conditional expression of Tert in pre-senescent cells (p21^high^ cells) could be used as a therapeutic tool to regulate AT remodeling and insulin-resistance in obesity. Very recently, it was reported that administration of a transcriptional activator of Tert in aged mice alleviates cellular senescence and systemic inflammation in different organs opening new avenues for therapeutic interventions [76]. *Tert* gene therapy in aged mice has been shown to delay aging without increasing the number of cancers [77]. However, long-term studies of p21^+/Tert^ mice show that 15% of these mice develop hepatocellular carcinomas, in part caused by the attenuation of senescence induced by the expression of Tert (unpublished data). The use of Tert for therapeutic purposes must thus be carefully controlled and restricted to the target tissue.

Finally, although our study focused on AT, the ubiquitous expression of p21-driven Tert raises the possibility that other metabolic organs, including liver, skeletal muscle, and pancreas may also contribute to the observed metabolic benefits. Future studies using tissue-specific models will be necessary to elucidate how these organs interact to influence systemic insulin sensitivity.

## 4 Materials and methods

### 4.1 Animals

Mice p21^+/−^ and p21^+/Tert^ were housed under controlled conditions of temperature (21 ± 1 °C), hygrometry (60 ± 10%) and lighting (12 h per day). Animals were acclimatized in the laboratory for one week before the start of the experiments. Mice were fed either a standard diet SD (A04, SAFE Diet, Augy, France) or a high fat diet HFD (60 kcal % fat, SAFE Diet, Augy, France). The SD used in this study was not fully nutrient-matched to the HFD, which may introduce minor differences beyond fat content. However, this approach aligns with standard practice employed in obesity models, allowing for meaningful comparison with the existing literature. All animals received care according to institutional guidelines, and all experiments were approved by the Institutional Ethics committee number 16, Paris, France (licence number 16–090). During follow-up, animals underwent body-weight, metabolic assessments. For EdU (EdU kit reagents Invitrogen C10646) treatments, mice were given two intraperitoneal injections of 5 mg/kg EdU in sterile PBS every 12 h two days before the sacrifice. Mice were euthanatized and organs and blood were collected and processed for further evaluations.

### 4.2 Fasting blood glucose, glucose and insulin tolerance tests

Whole-body glucose tolerance and insulin sensitivity were assessed in all groups at weeks 12th and 13th by intraperitoneal glucose (GTT) and insulin (ITT) tolerance tests, respectively. First, blood was obtained via tail clip to assess fasting blood glucose (Caresens® N, DinnoSanteTM). Then, mice received either glucose (1.5 g/kg) or insulin (0.3 UI/kg) solution (sigma I9278) by intraperitoneal injection, and blood glucose was measured at 15, 30, 60, 90 and 120 min after the injection. The area under the curve (AUC) for the glucose excursion was calculated using Graphpad Prism.

### 4.3 Plasma

Enzyme-linked immunosorbent assay (ELISA) kit (Life Technology Ref KMC2281) was used to measure Leptin level in the blood plasma.

### 4.4 Analysis of mRNA expression

For RNA extraction the samples were lysed with Qiazol (Qiagen, France) in the presence of chloroform, and total RNA was purified on mini-columns using RNeasy extraction kit (Qiagen, ref 74104). First-strand cDNA was synthesized from total RNA using the High-Capacity cDNA Reverse Transcription Kit (Life Technologies, ref4368814). Quantitative real-time PCR (qPCR) was performed in a StepOnePlus Real-Time PCR. Gene expression was assessed by the comparative CT (ΔΔCT) method with β-actin as the reference gene.

### 4.5 Histology and immunohistochemistry

Fresh visceral adipose tissue was fixed in 10% phosphate-buffered formalin overnight. Paraffin wax sections of 5 μm were prepared for immunostaining. Images of the haematoxylin-eosin-stained tissues were analyzed using Adiposoft Image J plugin (https://imagej.net/plugins/adiposoft). Click-EdU staining was performed on FFPE slides as described in the Invitrogen kit protocol (Click-EdU kit reagents Invitrogen C10646). See Supplemental Information, Methods for NF-κB and oxo-dG stainings.

### 4.6 Stromal vascular fraction (SVF) isolation and culture

Visceral fat was excised from the mice, minced with scissors, and digested for 1 h at 37 °C in 1 mg/ml collagenase type II digestion buffer (Life Technology, France; 17100017) in sterile Hank's Balanced Salt Solution (Life Technologies, ref 14185-052) containing 3% bovine serum albumin. After digestion, SVF were separated from adipocytes by centrifugation (300g, 3min) and two filtration steps (70 μm and 40 μm cell strainers). SVF were plated in a 6-well plate in DMEM supplemented with 0.5% Pen/Strep, 10% new born calf serum and 1% pyruvate.

### 4.7 Imaging flow cytometry analysis of the SVF

Purified SVF cells were incubated with a panel of antibodies (CD45 BD Bioscience 561047, CD31 BD Bioscience 612802, CD34 BioLegend 119314, CD29 BioLegend-102218, Sca1 BioLegend 108127, CD24 BioLegend-101823, PDGFRα BioLegend-135905, F4/80 BioLegend-123115, CD11b BD Bioscience-565976, CD11c BD Bioscience-749038, Live Dead Nir Cytek R7-60008) for 30 min on ice. Cells were then washed with 0.5% FBS/PBS FACS buffer and resuspended in 300 μL of FACS buffer. Cells were detected and their fluorescence was measured using Aurora flow cytometer (Cytek, Amsterdam, Netherland). Data analysis was performed in the SpectroFlow software.

### 4.8 Telomere Shortest Length Assay (TeSLA)

TeSLA (Telomere Shortest Length assay) is a method for measuring the distribution of the shortest telomeres in cells and tissues. TeSLA was performed according to the protocol described by [25]. Briefly, 50 ng of undigested genomic DNA was ligated with an equimolar mixture (50 pM each) of the six TeSLA-T oligonucleotides containing seven nucleotides of telomeric C-rich repeats at the 3′ end and 22 nucleotides of the unique sequence at the 5′ end. After overnight ligation at 35 °C, genomic DNA was digested with *Cvi*AII, *Bfa*I, *Nde*I, and *Mse*I, the restriction enzymes creating either AT or TA overhangs. Digested DNA was then treated with Shrimp Alkaline Phosphatase to remove 5′ phosphate from each DNA fragment to avoid their ligation to each other during the subsequent adapter ligation. Upon heat-inactivation of phosphatase, partially double-stranded AT and TA adapters were added (final concentration 1 μM each) and ligated to the dephosphorylated fragments of genomic DNA at 16 °C overnight. Following ligation of the adapters, genomic DNA was diluted to 20 pg/μL, and 2–4 μL was used in a 25 μL PCR reaction to amplify terminal fragments using primers complementary to the unique sequences at the 5’ ends of the TeSLA-T oligonucleotides and the AT/TA adapters. FailSafe polymerase mix (Epicenter) with 1 × FailSafe buffer H was used to amplify G-rich telomeric sequences. Entire PCR reactions were then loaded onto the 0.85% agarose gel for separation of the amplified fragments. To visualize telomeric fragments, the DNA was transferred from the gel onto the nylon membrane by Southern blotting procedure and hybridized with the ^32^P-labeled (CCCTAA)_3_ probe. The sizes of the telomeric fragments were quantified using TeSLA Quant software [25].

### 4.9 Cellular bioenergetic analysis Using the Seahorse bioscience XF analyzer

Bioenergetic profiles of the adipocytes were determined using a Seahorse Bioscience XF24 Analyzer (Billerica, MA, USA) that provides real-time measurements of oxygen consumption rate (OCR), indicative of mitochondrial respiration, and extracellular acidification rate (ECAR), an index of glycolysis as previously described [78].

### 4.10 Western blot analysis

Cells were lysed in cell lysis buffer (Cell Signaling, Danvers, MA France) supplemented with 1% phenylmethylsulfonyl fluoride (PMSF). Protein samples were separated on 12% bis-Tris gels and transferred to a nitrocellulose membrane. Primary antibodies against Akt and phospho-Akt (Ser473) were obtained from Cell Signaling (references #4991 and #4060 respectively) and the β-actin antibody was from Santa Cruz (reference sc-47778). Bands were visualized by enhanced chemiluminescence and quantified using ImageJ software.

### 4.11 Single-nuclei RNA sequencing

See Supplemental Information, Methods.

### 4.12 Statistical analysis

Data are expressed as mean values ± standard error of the mean (SEM). Statistical significance was tested using either one or two-way analysis of variance (ANOVA) with Fisher multiple comparison test. The results were considered significant if the p-value was <0.05.

## CRediT authorship contribution statement

**Laura Braud:** Writing – review & editing, Writing – original draft, Validation, Supervision, Software, Project administration, Methodology, Funding acquisition, Formal analysis, Conceptualization. **Manuel Bernabe:** Resources, Formal analysis, Data curation. **Julien Vernerey:** Visualization, Validation, Software. **Antonio M.A. Miranda:** Visualization, Validation, Software. **Andrea Dominguez:** Resources, Methodology, Formal analysis. **Dmitri Churikov:** Resources, Formal analysis. **Manon Richaud:** Validation, Software, Data curation, Conceptualization. **Frédéric Jourquin:** Validation, Resources. **Liam Mc Allan:** Validation, Software, Resources. **Christophe Lachaud:** Resources, Project administration, Formal analysis. **Jesus Gil:** Validation, Software, Resources, Methodology. **Will Scott:** Validation, Software, Resources. **Vincent Géli:** Writing – review & editing, Writing – original draft, Supervision, Resources, Project administration, Methodology, Funding acquisition, Conceptualization.

## Declaration of competing interest

J.G. has acted as a consultant for Unity Biotechnology, Geras Bio, Myricx Pharma Ltd., and Merck KGaA; owns equity in Geras Bio and share options in Myricx Pharma Ltd. and is a named inventor in MRC and imperial College patents related to senolytic therapies (unrelated to the work described here). J.G.’s lab received Pfizer and Unity Biotechnology unrelated to the work described here.

## Data Availability

Data will be made available on request.

## Appendix A Supplementary data

Fig S1 Tert expression does not impact metabolism of mice under standard diet and heterozygosity of p21 does not affect global metabolism of obese mice. A. Body weight (g) of male mice (n = 6 p21^+/−^ and n = 7 p21^+/Tert^ SD mice). B. Weight of the epididymal white adipose tissue (WAT) (g) (n = 6 p21^+/−^ and n = 7 p21^+/Tert^ SD mice). C. Glucose Tolerance Test (GTT) and area under the curve of the GTT (n = 6 p21^+/−^ and n = 7 p21^+/Tert^ SD mice). D. Insulin Tolerance Test (ITT) and area under the curve of the ITT (n = 6 p21^+/−^ and n = 7 p21^+/Tert^ SD mice). E. Weight acquisition kinetics of the indicated HFD mice (n = 8 p21^+/−^ HFD and n = 6 WT HFD mice). F. Epididymal white adipose tissue (WAT) weight of HFD mice at sacrifice (n = 8 p21^+/−^ HFD and n = 6 WT HFD mice). G. Liver weight (g) of mice at sacrifice (n = 8 p21^+/−^ HFD and n = 6 WT HFD mice). H. Glucose Tolerance Test (GTT) was performed by an intraperitoneal injection of glucose (1.5 g/kg) and measurement of glycemia via tail clip (Caresens® N, DinnoSanteTM) at different time points (n = 8 p21^+/−^ HFD and n = 6 WT HFD mice). I. Insulin Tolerance Test (ITT) was performed by an intraperitoneal injection of insulin (0.3 UI/kg) and glucose was measured via tail clip (Caresens® N, DinnoSanteTM) at different time points (n = 8 p21^+/−^ HFD and n = 6 WT HFD mice). Values represent the mean ± SEM. ∗p < 0.05 vs. p21^+/−^ SD mice (white bars). Student's t test or one-way ANOVA with Fisher multiple comparison test. Fig S2. Tert under the control of the p21 promoter does not impact female metabolism. A. Schematic representation of mouse models. B. Body weight (g) of female mice (n = 8 p21^+/−^ SD, n = 5 p21^+/−^ HFD and n = 8 p21^+/Tert^ HFD mice). C. Weight of the white epididymal adipose tissue (WAT) (g) (n = 8 p21^+/−^ SD, n = 5 p21^+/−^ HFD and n = 7 p21^+/Tert^ HFD mice). D. Leptin plasmatic level was measured by Leptin Elisa assay (n = 6 per group). E. Glucose Tolerance Test (GTT) and area under the curve of the GTT (n = 8 p21^+/−^ SD, n = 7 p21^+/−^ HFD and n = 6 p21^+/Tert^ HFD mice). F. Insulin Tolerance Test (ITT) and area under the curve of the ITT (n = 4 p21^+/−^ SD, n = 7 p21^+/−^ HFD and n = 7 p21^+/Tert^ HFD mice). G. p21 mRNA levels was measured by RT-PCR in stromal vascular fraction (SVF) of male and female (n = 8 p21^+/−^ SD male, n = 9 p21^+/−^ HFD male and n = 7 p21^+/Tert^ HFD male and n = 6 p21^+/−^ SD female, n = 6 p21^+/−^ HFD female and n = 6 p21^+/Tert^ HFD female mice). Values represent the mean ± SEM. ∗p < 0.05 vs. p21^+/−^ SD mice (white bars), #p < 0.05 vs. p21^+/−^ HFD (grey bars). Student's t test or one-way ANOVA with Fisher multiple comparison test. Fig S3. Tert expression does not impact ASPC of mice under standard diet. A. UMAP clustering of SVF cells. Cell populations were distinguished by FACS using the indicated markers. mCherry fluorescence was measured by FACS and signal quantification in all cells is indicated. (n = 3 mice per group). B. mCherry signal was measured by FACS. The percentage of mCherry positive cells is shown in the indicated populations of the SVF. (n = 3 p21^+/−^ SD, n = 3 p21^+/Tert^ SD mice). C. Hematoxylin and eosin (H&E) staining of visceral adipose tissue (WAT) from p21^+/−^ SD and p21^+/Tert^ SD mice. D. Adipocytes size in the images of H&E-stained WAT was quantified using AdipoSoft ImageJ software (n = 5 p21^+/−^ SD, n = 6 p21^+/Tert^ SD mice). E. Relative frequency of adipocyte sizes in the indicated groups of mice was plotted using GraphPad (n = 5 p21^+/−^ SD, n = 6 p21^+/Tert^ SD mice). F. Adipocyte cell density in the images of H&E-stained WAT was quantified using AdipoSoft ImageJ software (n = 5 p21^+/−^ SD, n = 6 p21^+/Tert^ SD mice). G. Representative flow cytometry data of ASPCs (pre-adipocytes and ASCs) of SVF from p21^+/−^ SD and p21^+/Tert^ SD mice. H. Relative percentage of ASCs in the WAT of the p21^+/−^ SD and p21^+/Tert^ SD mice analyzed by FACS (n = 5 p21^+/−^ SD, n = 3 p21^+/Tert^ SD mice). I. Relative percentage of pre-adipocytes in the WAT of the p21^+/−^ SD and p21^+/Tert^ SD mice analyzed by FACS (n = 5 p21^+/−^ SD, n = 3 p21^+/Tert^ SD mice). J. Mean telomere length of the telomeres detected by the Tesla method. (n = 3 per group). Values represent the mean ± SEM. ∗p < 0.05 vs. p21^+/−^ SD mice (white bars). Student's t test or one-way ANOVA with Fisher multiple comparison test. Fig S4. Adipose tissue stromal vascular fraction (SVF) subpopulation of cells analyzed by FACS. Gating strategy, Flow cytometry gating strategy used to define Immune Cells (CD45^+^ F4/80^-^), Leukocytes (CD45^+^ F4/80^-^ CD11b^−^ CD11c^−^), Myeloid cells (CD45^+^ F4/80^-^ CD11b^+^ CD11c^−^), Dendritic Cells type 1 (DC) (CD45^+^ F4/80^-^ CD11b^+^ CD11c^+^) and Dendritic Cells type 2 (DC-2) (CD45^+^ F4/80^-^ CD11b^−^ CD11c^+^), Macrophages (CD45^+^, F4/80^+^), MacCD11b^+^ (CD45^+^, F4/80^+^ CD11b^+^ CD11c^−^), MacCD11c^+^ (CD45^+^ F4/80^+^ CD11b^+^ CD11c^+^), Endothelial cells (CD45^-^ CD31^+^), Pericytes (CD45^-^ CD31^-^ CD34^+^ScaI^−^), Pre-adipocytes (CD45^-^ CD31^-^ F4/80^-^ CD11c^−^ CD11b^−^ CD34^+^ ScaI^+^ CD29^+^ PDGFRα^+^ CD24^-^) and Adipose Stem Cells (ASC) (CD45^-^ CD31^-^ F4/80^-^ CD11c^−^ CD11b^−^ CD34^+^ ScaI^+^ CD29^+^ PDGFRα^+^ CD24^+^). Dead cells were excluded with Live Dead Nir, cells were gated based on SSC-A versus FSC-A and singlets were selected from the SSC-A versus SSC-H dot plot. Fig S5. Analysis of short telomeres in the total WAT. A. Telomere Shortest Length Assay (TeSLA) of WAT using 30 pg of gDNA for each TeSLA PCR (n = 3 per group). B. Cumulative percentage of Tesla bands length bin (kb) (n = 3 per group). C. Mean telomere length of the telomeres detected by the Tesla method in the indicated groups of mice (n = 3 per group). Values represent the mean ± SEM. ∗p < 0.05 vs. p21^+/−^ SD mice (white bars), #p < 0.05 vs. p21^+/−^ HFD (grey bars). Student's t test or one-way ANOVA with Fisher multiple comparison test. Fig S6. Markers used for the subclustering of ASPC. A. Top unbiased markers of the different ASPC subtypes. Dotplots represent scaled expression per gene. B. Score of senescence signatures in all cell types, in adipocytes and in macrophages. C. Shared transcriptional signatures of ASPC subclusters with previously reported clusters Fig S7. Tert does not impact subcutaneous WAT. Expression of the genes involved in in lipogenesis, lipolysis and fatty acid oxidation was measured by RT-qPCR in SubCut WAT of the p21^+/−^ SD, p21^+/−^ HFD and p21^+/Tert^ HFD mice (n = 7 p21^+/−^ SD, n = 8 p21^+/−^ HFD and n = 8 p21^+/Tert^ HFD mice) Fig S1

Dataset 1 Number of cells for each cell type and for each condition Dataset 1

Dataset 2 Gene expression in ASPC analyzed by snRNAseq Dataset 2
