# Supplementary material for: TERT expression attenuates metabolic disorders in obese mice by promoting adipose stem and progenitor cell expansion and differentiation
Source: Mol Metab. 2025 Oct 3;102:102262. doi: 10.1016/j.molmet.2025.102262 (PMC12550303; doi:10.1016/j.molmet.2025.102262)
Supplement: Fig S1 — Tert expression does not impact metabolism of mice under standard diet and heterozygosity of p21 does not affect global metabolism of obese mice. A. Body weight (g) of male mice (n = 6 p21+/− and n = 7 p21+/Tert SD mice). B. Weight of the epididymal white adipose tissue (WAT) (g) (n = 6 p21+/− and n = 7 p21+/Tert SD mice). C. Glucose Tolerance Test (GTT) and area under the curve of the GTT (n = 6 p21+/− and n = 7 p21+/Tert SD mice). D. Insulin Tolerance Test (ITT) and area under the curve of the ITT (n = 6 p21+/− and n = 7 p21+/Tert SD mice). E. Weight acquisition kinetics of the indicated HFD mice (n = 8 p21+/− HFD and n = 6 WT HFD mice). F. Epididymal white adipose tissue (WAT) weight of HFD mice at sacrifice (n = 8 p21+/− HFD and n = 6 WT HFD mice). G. Liver weight (g) of mice at sacrifice (n = 8 p21+/− HFD and n = 6 WT HFD mice). H. Glucose Tolerance Test (GTT) was performed by an intraperitoneal injection of glucose (1.5 g/kg) and measurement of glycemia via tail clip (Caresens® N, DinnoSanteTM) at different time points (n = 8 p21+/− HFD and n = 6 WT HFD mice). I. Insulin Tolerance Test (ITT) was performed by an intraperitoneal injection of insulin (0.3 UI/kg) and glucose was measured via tail clip (Caresens® N, DinnoSanteTM) at different time points (n = 8 p21+/− HFD and n = 6 WT HFD mice). Values represent the mean ± SEM. ∗p < 0.05 vs. p21+/− SD mice (white bars). Student's t test or one-way ANOVA with Fisher multiple comparison test. Fig S2. Tert under the control of the p21 promoter does not impact female metabolism. A. Schematic representation of mouse models. B. Body weight (g) of female mice (n = 8 p21+/− SD, n = 5 p21+/− HFD and n = 8 p21+/Tert HFD mice). C. Weight of the white epididymal adipose tissue (WAT) (g) (n = 8 p21+/− SD, n = 5 p21+/− HFD and n = 7 p21+/Tert HFD mice). D. Leptin plasmatic level was measured by Leptin Elisa assay (n = 6 per group). E. Glucose Tolerance Test (GTT) and area under the curve of the GTT (n = 8 p21+/− SD, n = 7 p21+/− [file mmc1.pdf]

Suppl Fig 1.

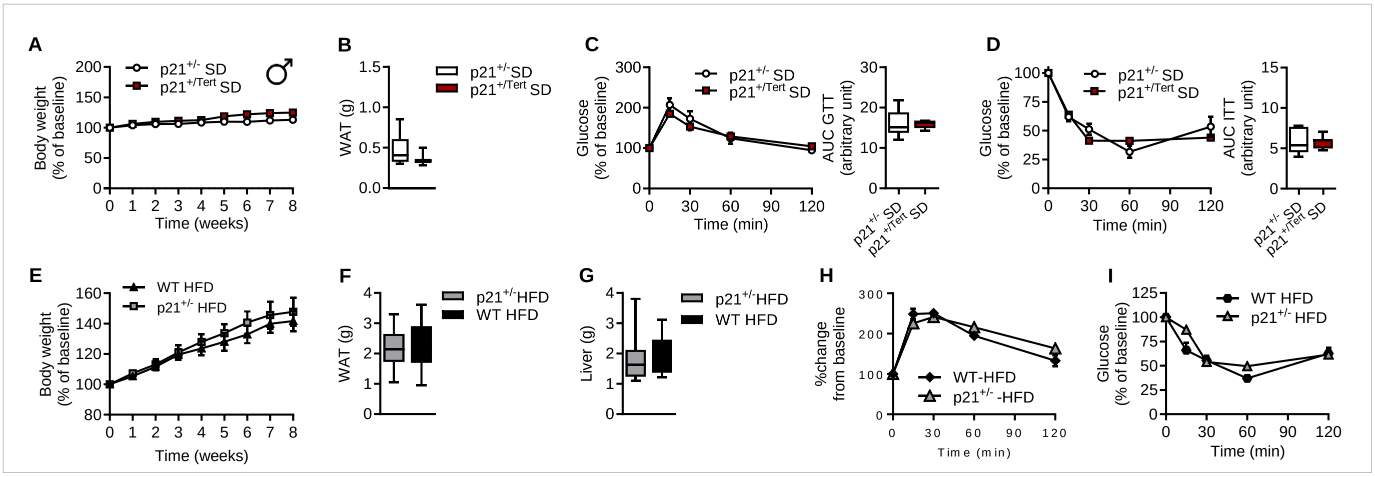

A
Body weight (% of baseline)
Time (weeks)
p21+/- SD
p21+/Tert SD
B
WAT (g)
p21+/-SD
p21+/Tert SD
C
Glucose (% of baseline)
Time (min)
p21+/- SD
p21+/Tert SD
AUC GTT (arbitrary unit)
D
Glucose (% of baseline)
Time (min)
p21+/- SD
p21+/Tert SD
AUC ITT (arbitrary unit)
E
Body weight (% of baseline)
Time (weeks)
WT HFD
p21+/- HFD
F
WAT (g)
p21+/-HFD
WT HFD
G
Liver (g)
p21+/-HFD
WT HFD
H
%change from baseline
Time (min)
WT-HFD
p21+/- -HFD
I
Glucose (% of baseline)
Time (min)
WT HFD
p21+/- HFD

Suppl Fig 2.

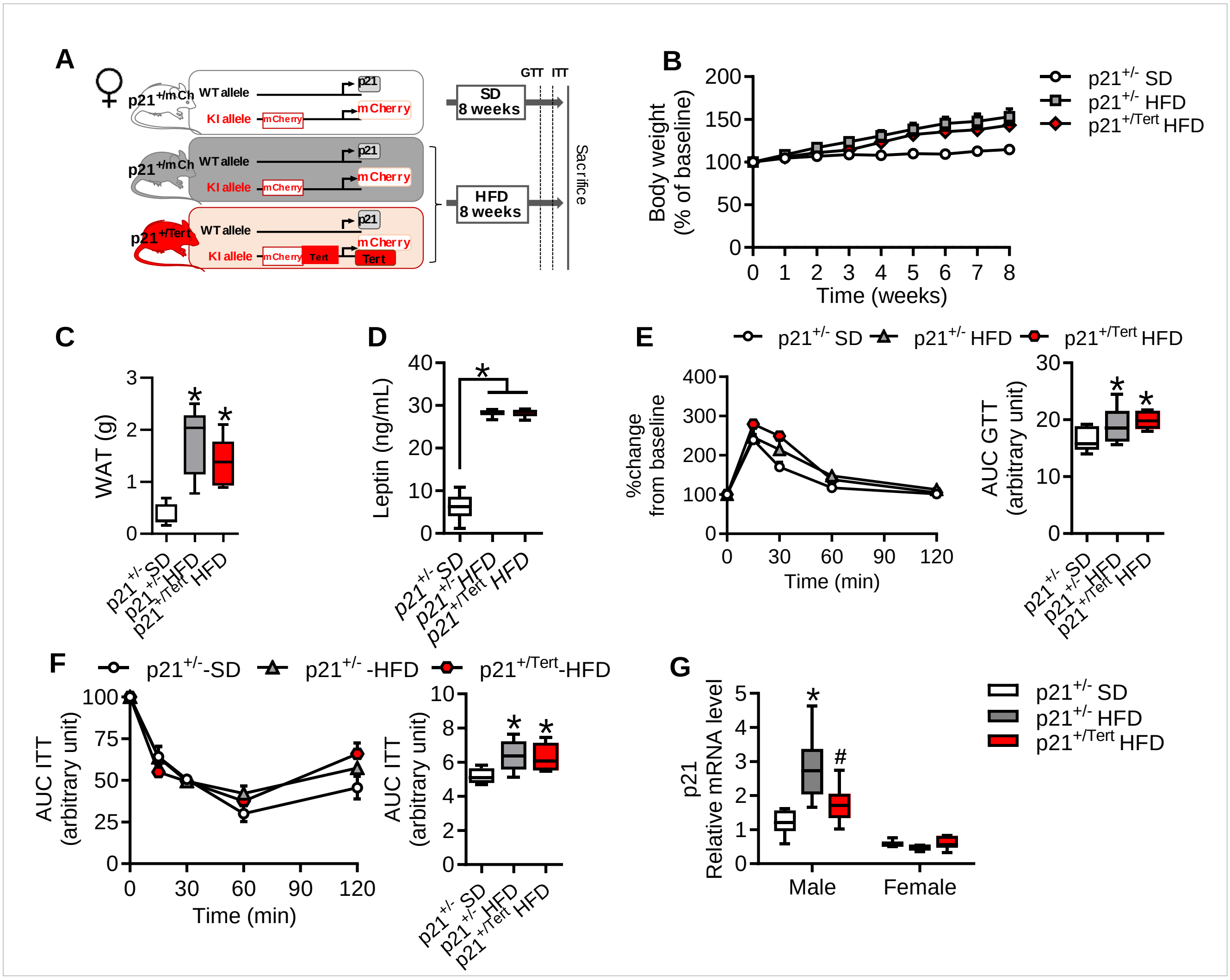
A
p21 +/mCh
WT allele
KI allele
mCherry
p21
p21 +/Tert
Tert
SD 8 weeks
HFD 8 weeks
GTT
ITT
Sacrifice
B
Body weight (% of baseline)
Time (weeks)
p21+/- SD
p21+/- HFD
p21+/Tert HFD
C
WAT (g)
D
Leptin (ng/mL)
E
%change from baseline
Time (min)
AUC GTT (arbitrary unit)
F
p21+/--SD
p21+/- -HFD
p21+/Tert-HFD
AUC ITT (arbitrary unit)
G
p21 Relative mRNA level
Male
Female

Suppl Fig 3.

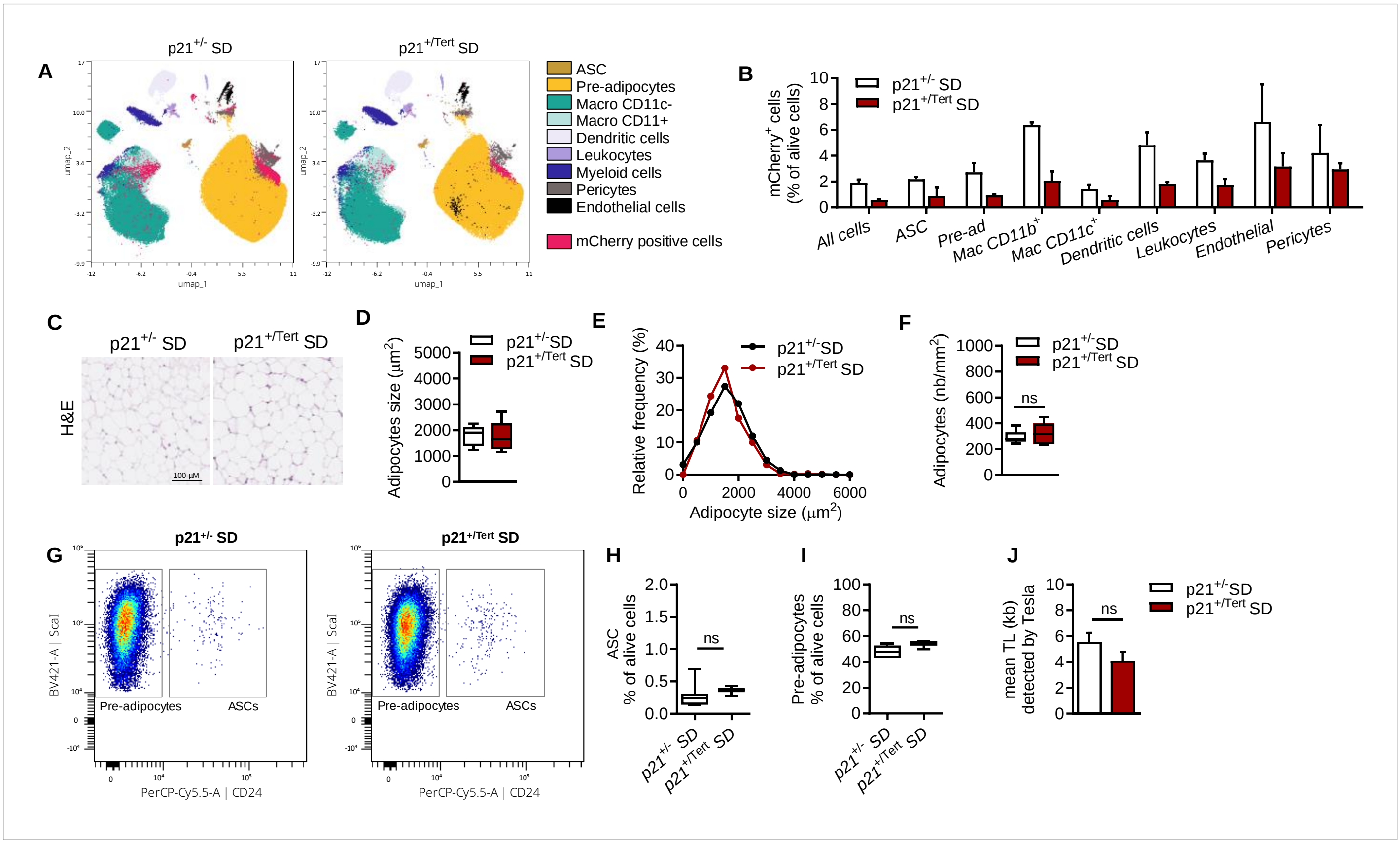

A
p21+/- SD
p21+/Tert SD
ASC
Pre-adipocytes
Macro CD11c-
Macro CD11+
Dendritic cells
Leukocytes
Myeloid cells
Pericytes
Endothelial cells
mCherry positive cells
umap_1
umap_2
B
mCherry+ cells (% of alive cells)
All cells
ASC
Pre-ad
Mac CD11b+
Mac CD11c+
Dendritic cells
Leukocytes
Endothelial
Pericytes
C
H&E
100 µM
D
Adipocytes size (µm2)
E
Relative frequency (%)
Adipocyte size (µm2)
F
Adipocytes (nb/mm2)
ns
G
BV421-A | ScaI
PerCP-Cy5.5-A | CD24
Pre-adipocytes
ASCs
H
ASC % of alive cells
I
Pre-adipocytes % of alive cells
J
mean TL (kb) detected by Tesla

Suppl Fig 4.

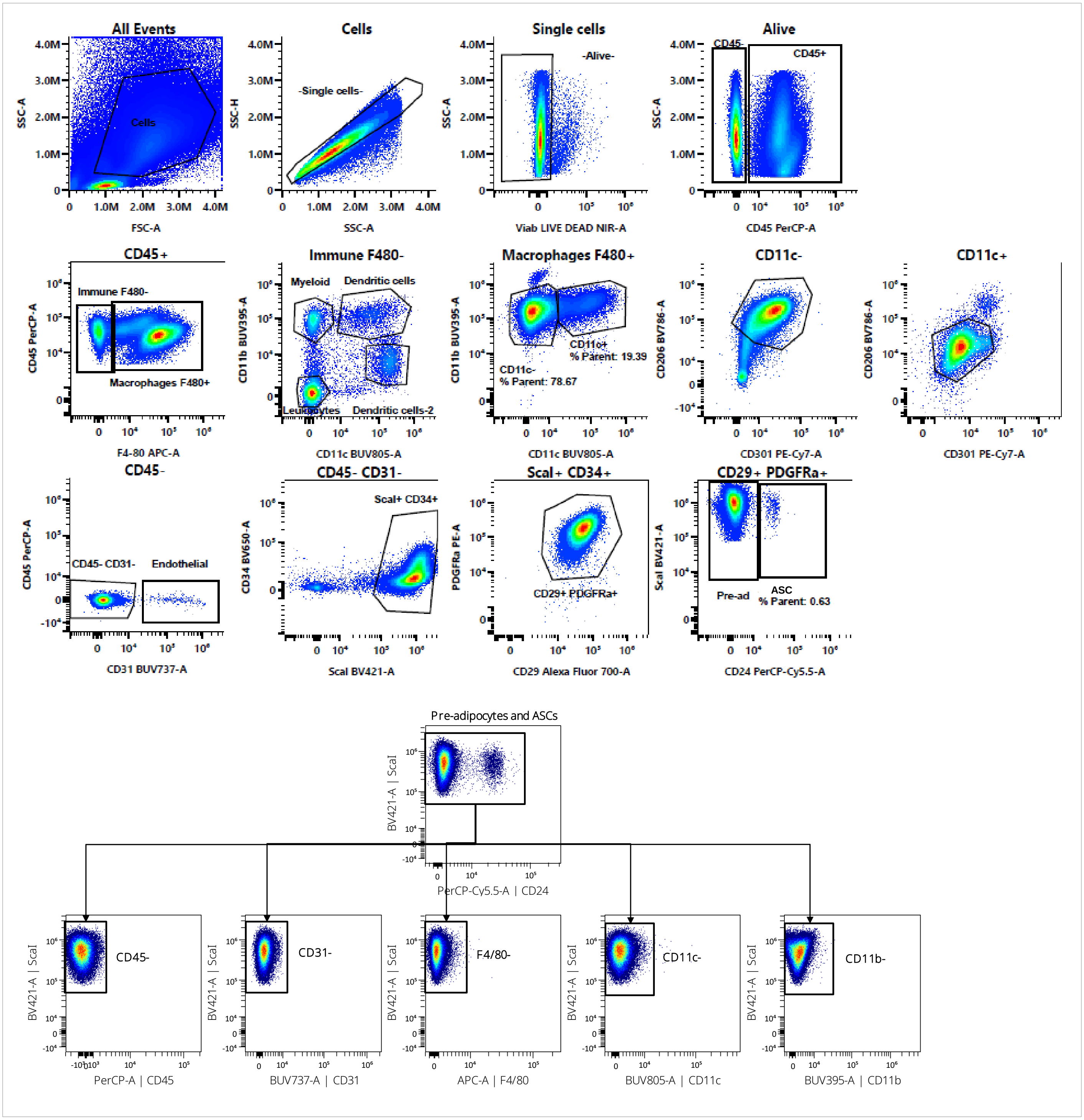

All Events
Cells
SSC-A
FSC-A
Single cells
-Single cells-
SSC-H
Alive
-Alive-
Viab LIVE DEAD NIR-A
CD45-
CD45+
CD45 PerCP-A
Immune F480-
Macrophages F480+
F4-80 APC-A
Myeloid
Dendritic cells
Leukocytes
Dendritic cells-2
CD11b BUV395-A
CD11c BUV805-A
CD11c+
% Parent: 19.39
CD11c-
% Parent: 78.67
CD206 BV786-A
CD301 PE-Cy7-A
CD45- CD31-
Endothelial
CD31 BUV737-A
Scal+ CD34+
CD34 BV650-A
Scal BV421-A
CD29+ PDGFRa+
PDGFRa PE-A
CD29 Alexa Fluor 700-A
Pre-ad
ASC
% Parent: 0.63
Scal BV421-A
CD24 PerCP-Cy5.5-A
Pre-adipocytes and ASCs
BV421-A | ScaI
PerCP-Cy5.5-A | CD24
CD45-
PerCP-A | CD45
CD31-
BUV737-A | CD31
F4/80-
APC-A | F4/80
CD11c-
BUV805-A | CD11c
CD11b-
BUV395-A | CD11b

Suppl Fig 5.

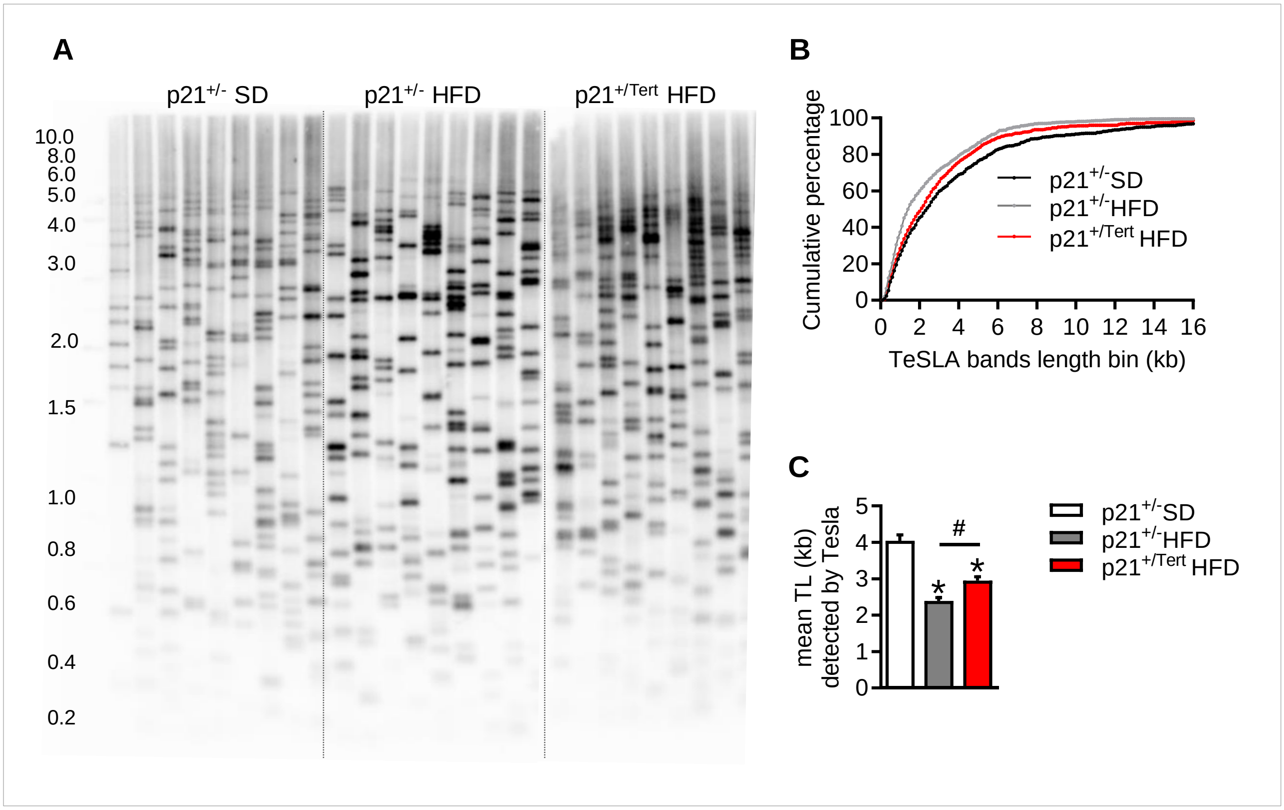
A
p21+/- SD
p21+/- HFD
p21+/Tert HFD
10.0
8.0
6.0
5.0
4.0
3.0
2.0
1.5
1.0
0.8
0.6
0.4
0.2
B
Cumulative percentage
100
80
60
40
20
0
0 2 4 6 8 10 12 14 16
TeSLA bands length bin (kb)
p21+/-SD
p21+/-HFD
p21+/Tert HFD
C
mean TL (kb) detected by Tesla
5
4
3
2
1
0
#
*
*
p21+/-SD
p21+/-HFD
p21+/Tert HFD

Suppl Fig 6.

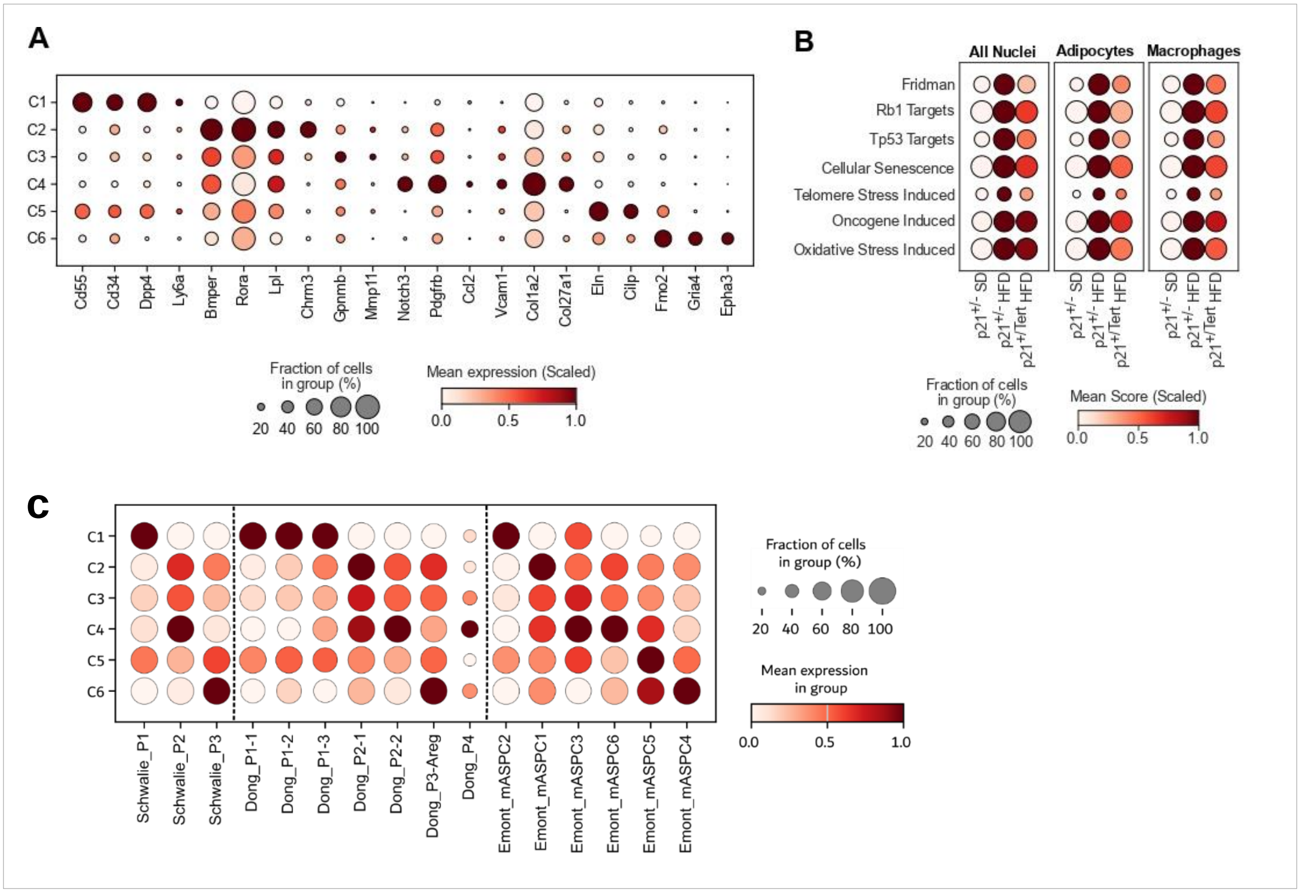
A
C1
C2
C3
C4
C5
C6
Cd55
Cd34
Dpp4
Ly6a
Bmper
Rora
Lpl
Chrm3
Gpnmb
Mmp11
Notch3
Pdgfrb
Ccl2
Vcam1
Col1a2
Col27a1
Eln
Cilp
Fmo2
Gria4
Epha3
Fraction of cells in group (%)
20 40 60 80 100
Mean expression (Scaled)
0.0 0.5 1.0
B
All Nuclei
Adipocytes
Macrophages
Fridman
Rb1 Targets
Tp53 Targets
Cellular Senescence
Telomere Stress Induced
Oncogene Induced
Oxidative Stress Induced
p21+/- SD
p21+/- HFD
p21+/Tert HFD
Fraction of cells in group (%)
20 40 60 80 100
Mean Score (Scaled)
0.0 0.5 1.0
C
Schwalie_P1
Schwalie_P2
Schwalie_P3
Dong_P1-1
Dong_P1-2
Dong_P1-3
Dong_P2-1
Dong_P2-2
Dong_P3-Areg
Dong_P4
Emont_mASPC2
Emont_mASPC1
Emont_mASPC3
Emont_mASPC6
Emont_mASPC5
Emont_mASPC4
Fraction of cells in group (%)
20 40 60 80 100
Mean expression in group
0.0 0.5 1.0

Suppl Fig 7.

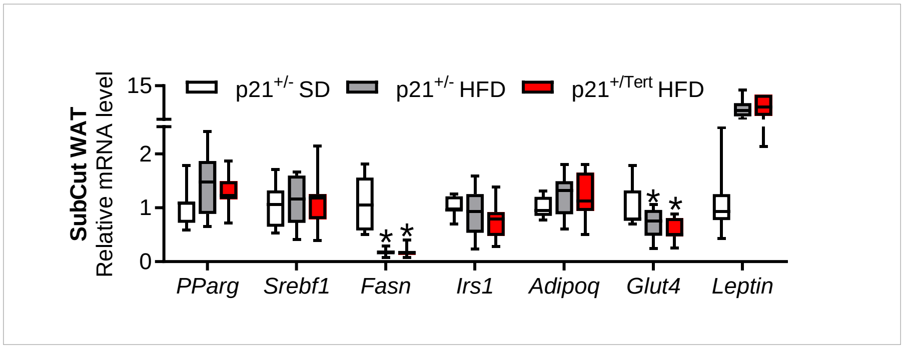

p21+/- SD
p21+/- HFD
p21+/Tert HFD
SubCut WAT
Relative mRNA level
15
2
1
0
PParg
Srebf1
Fasn
Irs1
Adipoq
Glut4
Leptin
